# The development and validation of prognostic models for overall survival in the presence of missing data in the training dataset: a strategy with a detailed example

**DOI:** 10.1186/s41512-021-00103-9

**Published:** 2021-08-04

**Authors:** Kara-Louise Royle, David A. Cairns

**Affiliations:** Clinical Trials Research Unit, Leeds Institute of Clinical Trials Research, University of Leeds, Leeds, UK

**Keywords:** Prognostic Model, Multiple imputation, Missing data, Overall survival

## Abstract

**Background:**

The United Kingdom Myeloma Research Alliance (UK-MRA) Myeloma Risk Profile is a prognostic model for overall survival. It was trained and tested on clinical trial data, aiming to improve the stratification of transplant ineligible (TNE) patients with newly diagnosed multiple myeloma. Missing data is a common problem which affects the development and validation of prognostic models, where decisions on how to address missingness have implications on the choice of methodology.

**Methods:**

Model building

The training and test datasets were the TNE pathways from two large randomised multicentre, phase III clinical trials. Potential prognostic factors were identified by expert opinion. Missing data in the training dataset was imputed using multiple imputation by chained equations. Univariate analysis fitted Cox proportional hazards models in each imputed dataset with the estimates combined by Rubin’s rules. Multivariable analysis applied penalised Cox regression models, with a fixed penalty term across the imputed datasets. The estimates from each imputed dataset and bootstrap standard errors were combined by Rubin’s rules to define the prognostic model.

Model assessment

Calibration was assessed by visualising the observed and predicted probabilities across the imputed datasets. Discrimination was assessed by combining the prognostic separation D-statistic from each imputed dataset by Rubin’s rules.

Model validation

The D-statistic was applied in a bootstrap internal validation process in the training dataset and an external validation process in the test dataset, where acceptable performance was pre-specified.

Development of risk groups

Risk groups were defined using the tertiles of the combined prognostic index, obtained by combining the prognostic index from each imputed dataset by Rubin’s rules.

**Results:**

The training dataset included 1852 patients, 1268 (68.47%) with complete case data. Ten imputed datasets were generated. Five hundred twenty patients were included in the test dataset. The D-statistic for the prognostic model was 0.840 (95% CI 0.716–0.964) in the training dataset and 0.654 (95% CI 0.497–0.811) in the test dataset and the corrected D-Statistic was 0.801.

**Conclusion:**

The decision to impute missing covariate data in the training dataset influenced the methods implemented to train and test the model. To extend current literature and aid future researchers, we have presented a detailed example of one approach. Whilst our example is not without limitations, a benefit is that all of the patient information available in the training dataset was utilised to develop the model.

**Trial registration:**

Both trials were registered; Myeloma IX-ISRCTN68454111, registered 21 September 2000. Myeloma XI-ISRCTN49407852, registered 24 June 2009.

**Supplementary Information:**

The online version contains supplementary material available at 10.1186/s41512-021-00103-9.

## Background

Multiple myeloma is a cancer of blood plasma cells of which there are 5000 new cases in the UK each year [1]. At diagnosis, patients are stratified into transplant eligible (TE) or transplant ineligible (TNE) groups by their treating clinician, determining whether they will receive high or low intensity treatment. Recent improvements in outcomes for patients with multiple myeloma have benefited TE patients over TNE patients [2]. There is a need to determine whether TNE patients can be stratified further in the context of their survival outcomes, so that treatment can be personalised and survival potentially improved.

A prognostic model can be used both to predict individuals’ risk of a future event and stratify patients. In the literature there are five areas commonly discussed concerning the development and validation of prognostic models: preliminary work, model building, model assessment, validation (internal and external) and the development of risk groups [3–6]. Preliminary work focuses on identifying both the data to develop the prognostic model (the training dataset) and the data in which to assess the developed model (the test dataset). It is also the stage when the variables (prognostic factors) to be considered for inclusion in the prognostic model are identified. Model building is concerned with developing the prognostic model, alongside deciding on how to handle any missing data. Model assessment considers how the developed prognostic model performs by investigating both the calibration (how well the predicted probabilities of the event of interest from the prognostic model match the empirical probabilities) and discrimination (whether those who experience the outcome of interest will be predicted to have it). Model validation considers both how the prognostic model performs in the training dataset (internal validation) and in the test dataset (external validation). Finally, the development of risk groups suggests how the prognostic model can be used to stratify patients into how likely they are to experience the outcome, which in this study was survival.

Missing data is not uncommon in the development of prognostic models and is an issue as it reduces the information available to build the model [7]. Rather than discard the observations or variables with missing information, it is suggested to impute any missing data, with sophisticated methods such as multiple imputation being advocated [4]. However, few published models implement imputation, particularly when considering survival outcomes [7–9]. Furthermore, the implications of multiple imputation on model development and validation are rarely discussed in publications, with the clinical impact of the model usually receiving more attention.

Here we aim to contribute to the literature by fully describing and discussing the methods implemented in a prognostic modelling study which was previously published according to the TRIPOD (Transparent reporting of a multivariable predication model for Individual Prognosis or Diagnosis) reporting guidelines [10]. The study aimed to train and test a prognostic model for overall survival in TNE patients with newly diagnosed multiple myeloma [11]. This example is chosen, as there was missing covariate data within the training dataset. The choice to impute this data influenced the implemented methods throughout the development and validation processes.

## Methods

Statistical analyses were largely undertaken in R: a language and environment for statistical computing (R Core Team, Vienna, Austria). SAS (version 9.4; SAS Institute, Cary, NC, USA) was used to organize the data and produce graphics where required.

### Preliminary work

The training dataset for this study was the non-intensive pathway of the National Cancer Research Institute (NCRI) Myeloma XI trial (ISRCTN49407852). Myeloma XI is a phase III trial in which newly diagnosed transplant ineligible patients with multiple myeloma were randomised to receive CTDa (attenuated cyclophosphamide, thalidomide, and dexamethasone) or CRDa (attenuated cyclophosphamide, lenalidomide, and dexamethasone) between 2010 and 2015. Full details of the trial design and results have been reported elsewhere [12, 13].

Clinical members of the Myeloma XI Trial Management Group proposed potential prognostic factors for overall survival to be considered for inclusion in the prognostic model. Each continuous variable was modelled as such, over the alternative approach of dichotomizing, to retain all the information they provided [3] and were assessed for normality using histograms and transformed where appropriate [14]. Each categorical variable was assessed to see if there was a natural ordering and it should be considered an ordinal variable. Ordinal variables were addressed using polynomial contrasts. These investigations and decisions were made so that the appropriate imputation model was selected for each covariate. This ensured that the functional form of the variables was specified at the start of the study.

It is suggested in the literature that a predictor which is highly correlated with another predictor will contribute little independent information and can therefore be excluded before variable selection [4]. In order to determine which variables should be included in the model building process, each pair of potential prognostic factors were compared graphically. Two continuous variables were compared using a scatter plot, two categorical variables were compared using a stacked bar chart and pairs of continuous and categorical variable were compared using a box plot. If two variables were considered to be highly correlated with each other, then only one of the variables was selected for the model building stage, according to clinical utility rather than statistical significance.

The preliminary work was conducted within R using the *ggplot2* package [15].

### Model building

To assess the extent of missing covariate data within the training dataset, the percentage of missing data within each variable was estimated. It was pre-specified that if no variable had a greater than 25% of missing data, ten imputed datasets would be generated [16]. Had this been violated, the number of imputed datasets would have equalled the proportion of missing data based on the commonly applied ‘rule of thumb’ [14] and the efficiency gained when increasing the number of datasets would have also been investigated [17].

Missing covariate data was imputed using multiple imputation by chained equations (MICE) as advocated in the literature [3]. The continuous variables were imputed using a linear regression model and predictive mean matching, whereas the ordinal variables were imputed using an ordinal regression model. Imputation was conducted in the order of variables with the least to the most missing data [14]. Each imputation model regressed the variable under consideration on the remaining five prognostic variables, the auxiliary variable sex (male or female), survival time in months and the survival indicator (death or censored). Despite the training data being taken from a clinical trial, randomised treatment allocation was not included in the imputation model. We made this decision because firstly, as treatment in myeloma is constantly evolving, including treatment as a proposed prognostic factor would limit the model’s applicability, and secondly the randomisation treatment for induction in Myeloma XI was not effective [13]. However, this decision might not be justified in a situation where there is a significant treatment effect. In addition, for simplicity, survival time in months was chosen to represent the survival outcome rather than the Nelson-Aalen estimator of the baseline cumulative hazard as recommended by White and Royston [18], as the aim here was not to focus on the imputation model, but the implication of imputation on the development and validation of the prognostic model.

As a penalised multivariable model was planned, following imputation the continuous variables were standardised within each imputed dataset using the mean and population standard deviation [19, 20]. In an imputed dataset of size *n* where *x*_*i*_ is the *i*^*th*^ observation of continuous variable *x* for *i* = 1, …, *n* with mean$\overline{x}$ , the standardised value (${x}_i^s$) was calculated as:

$$ {x}_i^s=\frac{x_i-\overline{x}}{\sigma },\mathrm{where}\ {\sigma}^2=\frac{\sum_i{\left({x}_i-\overline{x}\right)}^2}{n} $$

The standardisation was implemented to ensure that the continuous variables were penalised equally within the model building process. However, the ordinal variables were not standardised as they were modelled using polynomial contrasts and were therefore already being considered on a similar scale. In addition, it was thought that standardisation of the categorical variables would limit their interpretability in the estimated prognostic model and therefore they were not standardised.

Univariate analysis was conducted. This regressed each proposed prognostic factor on overall survival in a Cox proportional hazards model in turn. The univariate models were used to determine whether, when considered alone, the proposed prognostic factor was predictive of overall survival and for informal comparison with the multivariable model results, but, as per the guidance in the literature [4], not to determine which variables should be included in the multivariable model.

For multivariable analysis incorporating variable selection [4], a penalised Cox proportional hazards model was implemented. This statistical model is an extension of the standard Cox model in which an additional constraint is applied to the estimation of the parameters. For this analysis, the LASSO (least absolute shrinkage and selection operator) penalty term was applied and hence the parameter estimates were obtained by maximising the equation:

$$ \ell \left(\boldsymbol{\beta} \right)-\lambda {\sum}_i\left|{\beta}_i\right|, $$

where *ℓ*(***β***) represents the partial log-likelihood of the Cox model, *λ* the penalty term and ∣*β*_*i*_∣ the absolute value of the *i*^*th*^ parameter in the proposed model. The additional constraint, ∑_*i*_|*β*_*i*_|, results in some coefficients being shrunk to zero [19, 20].

The same penalty parameter *λ*^∗^ was applied within each imputed dataset to reduce the variability of variable selection across the imputed datasets [21]. The penalty parameter was obtained as follows. First, the optimum value of the penalty parameter within each imputed dataset was derived using a cross-validation process as proposed by Simon et al. [22]. The mean of the optimum values across the imputed datasets was calculated and defined as the penalty parameter (*λ*^∗^) for the model building process. Note that the lambda sequence for the cross-validation process was obtained from the first iteration of cross-validation.

A penalised Cox model that regressed overall survival on the potential prognostic factors was estimated in each imputed dataset with penalty parameter *λ*^∗^. In addition, as standard errors are not readily interpretable in a penalised model [23], bootstrap standard errors for the coefficients were derived using 100 resamples within each imputed dataset.

The prognostic model was obtained by combining the coefficients and bootstrap standard errors from each imputed dataset by Rubin’s rules [16]. If a variable was included in a subset of the models derived, the total number of imputed datasets, ten, was used as the denominator in Rubin’s rules to combine the results together.

The following R packages were used during the model building process; *mice* [24], *survival* [25], *hdnom* [26], and *glmnet* [27]. To enable the packages to work together, code was written by KLR to derive the bootstrap standard errors and combine the multivariable model results using Rubin’s rules.

### Model assessment

We assessed model performance in each imputed dataset by considering the assumptions of the penalised Cox model, calibration and discrimination. The final model assessment was obtained by combining or summarising the results across the imputed datasets.

#### Model assumptions

The assumption of proportional hazards was tested in each imputed dataset by regressing the weighted Schoenfeld residuals on time in a linear regression model for each covariate in turn instead of the more commonly used scaled Schoenfeld residuals [28]. This was to account for the possibility that the bootstrap variance-covariance matrix would be singular. Proportional hazards for a covariate were concluded if the regression coefficient for time was not significantly different from zero at the 5% significance level. If the proportional hazards assumption had been violated, time-dependent covariates and other more flexible survival models would have been considered [29].

The above was implemented using code written by KLR.

#### Calibration

In order to assess the calibration of the prognostic model, the predicted and observed probabilities were calculated in each imputed dataset at 60 days and 1 year, using an approach similar to that described by Harrell [3].

The predicted probabilities were obtained by applying the model derived within the imputed dataset to the data. The quantiles of the predicted probabilities were used to split the data into ten risk groups. The predicted probability for a risk group was taken to be the median of the predicted probabilities of individuals included in the risk group. The observed probability for a risk group was calculated using the Kaplan-Meier method [26].

The calibration of the prognostic model was assessed by plotting the predicted vs observed probability plots for each imputed dataset. This method was chosen as the preferred shrinkage estimate could not be easily combined using Rubin’s rules to obtain an overall measure of calibration performance [30]. Therefore, it was decided to assess calibration graphically rather than with a single estimate.

The above was implemented using the package *hdnom* [26] and *ggplot2* [15].

#### Discrimination

To assess the discrimination of the prognostic model, it was first assessed in each imputed dataset using the prognostic separation D-statistic (D-statistic). The D-statistic was used as, unlike other measures, it could be calculated within each imputed dataset and then combined via Rubin’s rules without the need for any robust methods [30].

The D-statistic is defined as the slope coefficient of a model for overall survival on the scaled rankits [31]. It was calculated in each imputed dataset (of sample size *n*) by applying the following steps:

I. The prognostic index (linear predictor) was calculated for each individual
II. The values calculated in I were ordered from smallest to largest and assigned a position (*i* = 1, …, *n*)
III. The scaled rankits (*z*_*i*_) were calculated using Bloom’s approximation [31]:
  $$ {z}_i={\left(\frac{8}{\pi}\right)}^{-\frac{1}{2}}{\varPhi}^{-1}\left(\frac{i-\frac{3}{8}}{n+\frac{1}{4}}\right) $$
IV. The scaled rankit, *z*_*i*_, was assigned to the individual in position *i*, i.e. the smallest scaled rankit was assigned to the individual with the smallest prognostic index
V. Overall survival was regressed on the scaled rankits in a Cox regression model
VI. The regression coefficient of the scaled rankits was defined as the D-statistic

The D-statistic of the prognostic model was estimated by combining the results in each imputed dataset using Rubin’s rules. This quantity is referred to as the combined D-statistic.

The above was implemented using the *survival* [25] package in conjunction with code written by KLR to calculate the D-Statistic and combine the results using Rubin’s rules.

### Model validation

#### Internal validation

So that all of the data in the training dataset could be used within the model building process, a bootstrap internal validation process was implemented [3]. Discrimination performance in terms of the D-statistic was used to measure acceptable performance for the internal validation process. To ensure that the approach of multiple imputation was accounted for within the validation process [21], the internal validation process was implemented using the following steps:

I. A sample with replacement was drawn from the incomplete training data
II. The model building process detailed above, including the imputation, was repeated to give ten imputed datasets, ten models and ten D-statistics
III. The results of step II were combined using Rubin’s rules to give a sample prognostic model and a sample D-statistic
IV. The sample prognostic model was applied to the ten imputed datasets obtained in the original model building process and a D-statistic was obtained
V. The D-statistics obtained in step III were combined using Rubin’s rules and the result referred to as the internal validation D-statistic
VI. The difference between the internal validation D-statistic and sample D-statistic was calculated and defined as the optimism
VII. Steps I–VI were repeated 100 times
VIII. The mean optimism across the 100 iterations was estimated
IX. The corrected D-statistic was estimated as the D-statistic obtained in the model assessment minus the mean optimism

The internal validation process was implemented using code written by KLR which incorporated each element of the model building process.

#### External validation

In order to determine how well the developed model performed in data different to which it was built, an external validation process was conducted using the non-intensive pathway of the Medical Research Council (MRC) Myeloma IX trial (ISRCTN68454111) as the test dataset. Myeloma IX is a phase III trial in which newly diagnosed transplant ineligible patients with multiple myeloma were randomised to receive CTDa or MP (melphalan and prednisolone) between 2003 and 2007. Full details of the trial design and results have been published elsewhere [32]. As the model was aimed at being applied in clinic, individuals in Myeloma IX were included in the test dataset if they had all the variables included in the prognostic model available, i.e. only individuals with complete case data were included in the test dataset.

With the exception of imputation, the test dataset was treated similarly to the training dataset, i.e. the transformations applied to the continuous variables in the training dataset were applied to those in the test dataset. The continuous variables were also standardised using values estimated in the model building process combined using Rubin’s rules. The prognostic index of the test model was obtained using the coefficients from the prognostic model derived in the training dataset.

Both the calibration and discrimination of the prognostic model were assessed in the test dataset using the measures described above.

Both the assessment of calibration and discrimination was implemented using code written by KLR. The code for calibration was written such that the process was equivalent to that of *hdnom* [26].

### Risk group development

In order for the clinical community to stratify patients easily, risk groups were derived. To account for the imputation process, the prognostic indexes from the models built in the ten imputed datasets were combined using Rubin’s rules to derive the combined prognostic index. The tertiles of the combined prognostic index, calculated within the training dataset, were used to trichotomize individuals in both the test and training datasets into the risk groups: low, medium and high. The risk groups were used to stratify Kaplan-Meier curves of overall survival in both the test and training datasets.

The risk groups were derived in R using code written by KLR. Kaplan-Meier curves were plotted in SAS.

### Additional analysis

Following the implementation of the above methods, additional analysis and sensitivity analysis was conducted to investigate some of the identified limitations.

#### Model building and assessment

As the main analysis assessed discrimination using the lesser known D-statistic, Uno’s C-statistic [33] was calculated at both 60 days and 1 year.

In addition, to extend the assessment of calibration, a regression model was fitted within each imputed dataset. The regression model regressed the predicted probability estimates on the observed probability estimates from which an estimate of calibration slope was obtained for both 60 days and 1 year. The ten estimates of calibration slope were then combined using Rubin’s rules to obtain a single estimate. Note that as only ten estimates were used in the regression analysis, confidence intervals are not presented for this estimate.

#### Model validation—calibration

The internal and external validation processes described above were both extended to consider calibration using the measurement of calibration slope. However, as this work was not prospective, acceptable performance in terms of calibration was not pre-specified.

Internal validation was conducted using an identical process to that described for discrimination. The external validation process applied the developed model to the test dataset and calculated the predicted and observed probabilities at 60 days and 1 year using similar methods to that in the training dataset. From this, estimates of calibration slope were obtained.

#### Model validation—external validation

A sensitivity analysis was conducted which imputed missing data in the test dataset. To remain consistent with model development, ten imputed datasets were created using the same imputation models. The final combined model from the development process was applied within each imputed dataset and both calibration and discrimination were assessed in the same way as in the pre-specified analysis.

With the exception of the UnoC command, within the SurvAUC package [34], to calculate Uno’s C-statistic, no additional packages were used in the additional analysis to that described in the main analysis.

## Results

### Preliminary work

The training dataset for this study was formed of the 1852 TNE patients in Myeloma XI. Median follow-up was 32.7 months (inter-quartile range (IQR) 17.7–47.3 months). Six routinely collected patient characteristics and measurements of disease were proposed by clinicians as potential prognostic factors: age, WHO (world health organisation) performance status (PS), lactate dehydrogenase (LDH), C-reactive protein (CRP), international staging system (ISS) and the ratio of lymphocytes to total white blood cells (L:W) (Table 1). Of the 1852 participants in the training dataset, 1268 (68.47%) had all six potential prognostic factors recorded and 700 (37.80%) had died at the time of analysis. Table 2 shows the missing data pattern for the six variables in the training dataset. Of the six variables, LDH had the largest proportion of missing data with 22.46% of participants not having a value recorded.

**Table 1:** Potential prognostic factors considered for inclusion within the prognostic model

Potential prognostic factor	Description	Type
Age	Age derived at trial registration	Continuous
WHO PS	World Health Organisation Performance Status, a measure of the patient’s ability to do daily tasks	Ordinal range from 0 (alive and fully active) to 5 (dead)
LDH	Lactate dehydrogenase level in the blood, a measure of tissue damage	Continuous
CRP	C-reactive protein level in the blood, a measure of inflammation	Continuous
L:W	Lymphocyte count to total white blood cell count ratio	Continuous
ISS	International Staging System, a measure of the severity of disease in multiple myeloma calculated dependent on the levels of β2-microglobulm and albumin in the blood	Ordinal range from stage I to stage III

**Table 2:** Missing data pattern in the training dataset. The percentage of missing data for a single proposed prognostic factor within the training dataset can be calculated by summing all the patterns that include that variable

Pattern	n (%)
Complete cases	1268 (68.47)
LDH	187 (10.10)
LDH and CRP	107 (5.78)
CRP	74 (4.00)
ISS	46 (2.48)
ISS and LDH and CRP	30 (1.62)
ISS and LDH	28 (1.51)
WHO PS	27 (1.46)
WHO PS and LDH	20 (1.08)
WHO PS and LDH and CRP	16 (0.86)
WHO PS and ISS and LDH and CRP	13 (0.70)
ISS and CRP	9 (0.49)
WHO PS and ISS and LDH	9 (0.49)
WHO PS and CRP	7 (0.38)
L:W and WHO PS and ISS and LDH and CRP	5 (0.27)
WHO and ISS	4 (0.22)
L:W	1 (0.05)
L:W and LDH and CRP	1 (0.05)
Age	0 (0.00)

Histograms of CRP and LDH (Additional File 1) showed skewed distributions and resulted in the two variables being transformed using the log + 1 and log functions respectively. As a result of their natural ordering, WHO PS and ISS were considered as ordinal variables and modelled using polynomial contrasts. Plots of each pair of potential prognostic factors showed that no two variables were strongly correlated. Therefore, all six variables were considered for inclusion in the prognostic model (Additional File 2).

### Model building

**Fig. 1:**
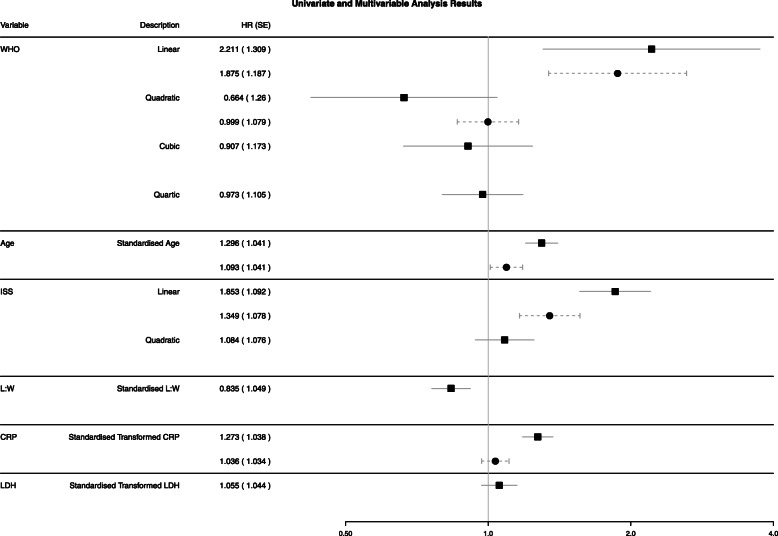
Results of the univariate and multivariable analysis conducted within the training dataset. The black squares and solid horizontal lines represent the estimate and the associated 95% confidence interval of the proposed prognostic model within a univariate Cox model. The black circles and dashed horizontal lines represent the estimate and the associated 95% confidence interval of the proposed prognostic model within a multivariable penalised Cox model, following the combination of coefficients and bootstrap standard errors across ten imputed dataset using Rubin’s rules. If there are no multivariable results for a variable, then this was penalised to zero during the model building process and not included in the final model

Ten imputed datasets were obtained. Univariate analysis suggested that when considered alone all the prognostic variables, with the exception of LDH, were significantly associated with overall survival (Fig. 1).

The mean of the ten ‘optimal’ penalty parameters was 0.0544 (standard deviation (SD): 0.00162), indicating little variation across the imputed datasets. The penalty parameter of 0.0544 resulted in some variation across the variables selected for inclusion in the model across the imputed datasets, where the quadratic term of WHO PS was only included in one out of ten models. The remaining variables were selected in all or none of the ten models. The final prognostic model included the linear and quadratic elements of WHO PS, the linear term of ISS, age and CRP (Fig. 1).

### Model assessment

None of the linear regression models for each covariate within each imputed dataset concluded that the regression coefficient for time was significantly different from zero. Therefore, no evidence was found to suggest the proportional hazards assumption was violated.

The calibration of the prognostic model was better at 60 days post randomisation compared to one-year post randomisation (Additional File 3). There was little variation across the D-statistics calculated in each imputed dataset (within imputation variance 0.00393, across imputation variance 0.0000598). The combined D-statistic was 0.840 (95% confidence interval (95% CI) 0.716–0.964).

### Internal validation

**Fig. 2:**
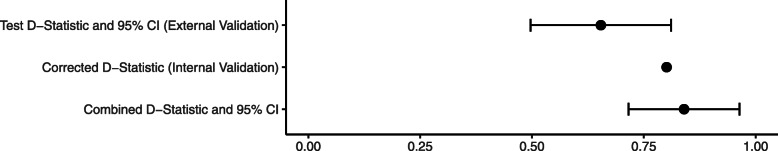
Results of the internal and external validation processes. The points represent the point estimates of the various discrimination measures and the horizontal lines show the 95% confidence intervals, where appropriate

The mean optimism from the internal validation process was 0.039 (SD: 0.0639). The corrected D-statistic was 0.801, resulting in the prognostic model being concluded to perform acceptably in the training dataset (Fig. 2).

### External validation

Myeloma IX recruited 849 TNE patients. Of these, 520 (61.25%) had WHO PS, ISS, age and CRP recorded and were included in the test dataset. In the test dataset, 411 (79.04%) participants had died at the time of the analysis. Median follow-up of the test dataset was 69.2 (IQR 56.0–83.2) months. When applied, the model performed similarly in terms of calibration, with the majority of confidence intervals around the observed estimate crossing the identity line at 60 days compared to only a few at 1 year (Additional File 3) and discrimination where the estimates of the D-statistics are similar and 95% confidence intervals around the D-statistics in both the training and test datasets overlap somewhat (Fig. 2).

### Development of risk groups

**Fig. 3:**
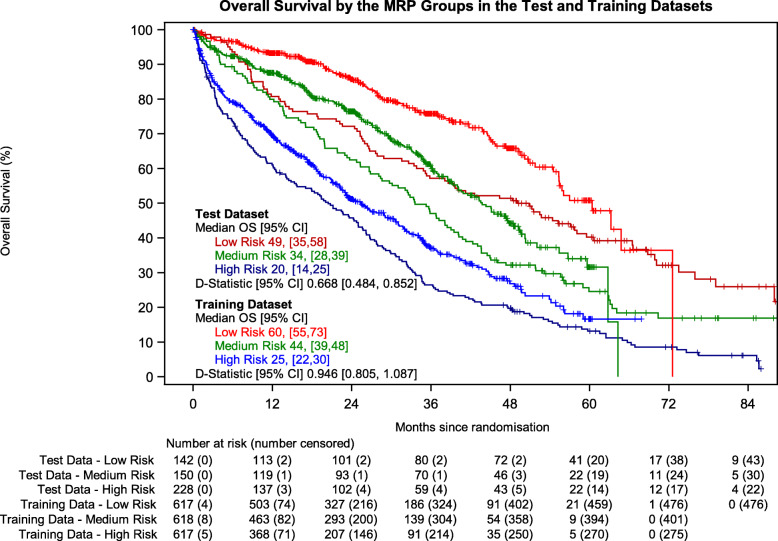
Kaplan-Meier curves for overall survival by the derived risk groups in the test and training datasets

The tertiles of the combined prognostic index were − 0.256 and 0.0283. This resulted in 617 (141 events), 618 (217 events) and 617 (342 events) participants being categorised as low, medium and high-risk in the training dataset and 142 (90 events), 150 (115 events) and 228 (202 events) in the test dataset. Figure 3 shows the Kaplan-Meier estimate of the survival function for overall survival stratified by the risk groups in both the training and test datasets.

### Additional analysis—model assessment and validation

The median (IQR) of Uno’s C-statistic across the ten imputations of the training dataset was 0.665 (0.661–0.667) at 60 days and 0.680 (0.679–0.681) at 1 year. In the test dataset, the point estimates were 0.712 and 0.631 at 60 days and 1 year respectively.

The combined estimate of calibration slope was 2.10 at 60 days and 2.43 at 1 year in the training dataset and 5.44 at 60 days and 2.63 at 1 year in the test dataset.

The average optimism across the one hundred bootstrap samples was − 3.65 for 60 days and − 2.47 for 1-year post-randomisation. The corrected calibration slopes were therefore 5.74 and 4.90, respectively.

### Additional analysis—multiple imputation sensitivity analysis

The test dataset for the sensitivity analysis considering multiple imputation included all 849 TNE patients from Myeloma IX. At the time of analysis, 651 participants had died. Median follow-up was 68.8 (IQR 56.1–82.0). Table 3 shows the missing data pattern for the six variables in the test dataset. Of the six variables, LDH had the largest proportion of missing data with 44.41% of participants not having a value recorded.

**Table 3:** Missing data pattern in the test dataset. The percentage of missing data for a single proposed prognostic factor within the test dataset can be calculated by summing all the patterns that include that variable

Pattern	n (%)
Complete cases	361 (42.52)
LDH	157 (18.49)
LDH and CRP	140 (16.49)
CRP	91 (10.72)
ISS and LDH	42 (4.95)
ISS and LDH and CRP	33 (3.89)
ISS	10 (1.18)
ISS and CRP	7 (0.82)
LW ratio and LDH	2 (0.24)
WHO PS and LDH and CRP	2 (0.24)
LW ratio and CRP	1 (0.12)
LW ratio and ISS	1 (0.12)
WHO PS and ISS and LDH and CRP	1 (0.12)
WHO PS and CRP	1 (0.12)
Age	0 (0)

The predicted versus observed probability plots at 60 days and 1 year are shown in Additional File 4. The combined estimate of calibration slope was 5.85 at 60 days and 3.10 at 1 year. The combined D-statistic (95% CI) was 0.666 (0.536–0.795).

## Discussion

**Fig. 4:**
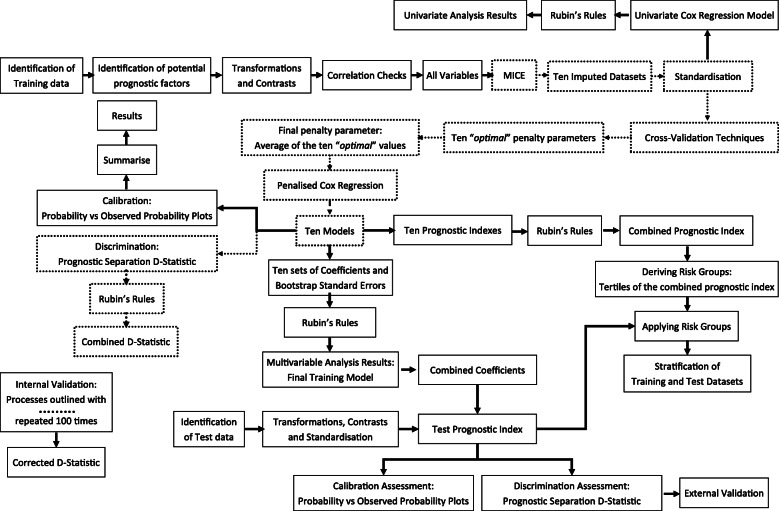
An overview of the implemented methods. The dashed border represents the steps repeated from the model building process in the internal validation process

Here we have described in detail the methods implemented during both the development and validation processes of a prognostic model for a survival outcome, the clinical impact of which has been discussed previously [11]. Figure 4 is an overview of the implemented methods and shows how the processes in the development and validation of a prognostic model interlink. The decision to use these methods and choices on how they were implemented were driven by the existing guidance on the development and validation of prognostic models [3–5] and also by their viability of application in conjunction with multiple imputation.

The training dataset of this study was a subset of a large phase III randomised controlled trial. The data was chosen as it was believed to be representative of the population of interest. However, as the proposed prognostic factors were not stratification factors, there was missing data present in five out of the six variables. Missing data can be categorised as three mechanisms: missing completely at random (MCAR), missing at random (MAR) and missing not at random (MNAR) [35]. The mechanism assumed determines whether a complete case analysis is appropriate. For example, under the MCAR assumption a complete case analysis is appropriate. In addition, for MAR and MNAR, a complete case analysis may be appropriate if missingness does not depend on outcome [36]. However, in either case, it may not be optimal due to the reduction in sample size. In this study, all of the proposed variables were patient characteristics and measurements of disease, which are routinely measured as part of standard practice prior to a patient starting treatment. In the case of myeloma patients, it is likely that one of these variables is missing due to another similar test being performed instead. Because of this, we assumed the data to be MAR rather than MNAR. As such, missing covariate data was imputed using MICE. MICE was chosen as it is recommended for use in the literature and because it involves multiple datasets being created, meaning that uncertainty is considered in the process compared to single imputation methods such as mean imputation [37]. If a complete case analysis or single imputation method had been deemed appropriate, the limitations around model assessment would have been avoided, with the commonly used measurements of calibration and discrimination easily applicable and available to assess acceptable model performance in both the training and test datasets.

The combination of multiple imputation and penalised Cox regression raises the question of how best to combine results across imputed datasets when some models include a variable and some do not. The approach taken here is the same as that taken by Musoro et al. [21] where despite variables being penalised and excluded in one or more models across the imputed datasets, the number of imputed datasets in the calculations of Rubin’s rules remains the same. From our perspective, this is appropriate as it represents that penalisation has occurred in some, but not all, of the imputed datasets.

The main challenge of this work was appropriately assessing how the developed model performed in terms of discrimination and calibration. Throughout the literature, there are many ways in which one can assess calibration or discrimination, the most common being the C-statistic for discrimination and the shrinkage estimate for calibration. However, for both of these measures, the normality assumption required under Rubin’s rules is uncertain and robust methods are required to obtain a single estimate across the imputed datasets. Alternatively, summarising these results over the imputed datasets using the median and interquartile range may also be appropriate [30]. In this analysis, we assessed discrimination using the lesser-used D-statistic because it can be combined easily using Rubin’s rules. However, on reflection and to aid reader interpretation, we have added Uno’s C-statistic at both 60 days and 1 year in the training and test datasets. Initially in this work, we assessed calibration visually, using predicted versus observed probability plots. From this, we concluded that the model was likely over estimating the true probabilities. However, we decided that calibration was acceptable, as the confidence intervals cross the desired observed probability equals predicted probability line for the majority of risk groups, across the imputed datasets in the training dataset and in the test dataset (Additional File 3). We have subsequently investigated the measurement of calibration slope as an assessment of calibration. This supported our initial findings that the model was over-estimating the observed probabilities, as the combined estimate of calibration slope was greater than two at both timepoints, in both datasets. However, the assessment of calibration in survival models is not simple. The derivation of risk groups reduces the number of points used in any regression equation thus raising questions of model misspecification and is the reason why precision estimates were not presented for this measure. In addition, the measurement of calibration slope only considers the point estimates from the regression model whereas variation in the estimates should be considered. We believe that further work is required to determine how best to assess calibration in the context of survival models in the presence of missing data.

The integration of multiple imputation and internal validation has been considered in the literature [21, 38, 39]. Vergouwe et al. [38] did not include internal validation in their methods, but questioned in the discussion which should come first: imputation or drawing a bootstrap sample. Musoro et al. [21] developed this and considered whether imputing prior to taking the bootstrap sample appropriately estimated the optimism. Wahl et al. [39] developed this further and investigated through simulation studies and real data examples the implications of different orderings of imputation and a variety of resampling methods. Both investigations recommend drawing a bootstrap sample first then performing imputation. The method applied here is closer to that conducted by Musoro rather than Wahl as the former included additional considerations around the inclusion of a penalty parameter in the multivariable model.

In contrast to model building and internal validation, external validation was conducted using a complete case approach. In the literature, multiple imputation has been applied in the test dataset and the same methods could have been considered here [38]. However, for the analysis of primacy, it was felt that the external validation process should be representative of what will likely occur in clinical practice— where if one variable is missing, a score is not calculated. However, multiple imputation was applied in a sensitivity analysis. In this analysis, the developed model performed similarly. However, a validated and regulated process is required before real-time imputation occurs in clinic. In the interim, we would recommend that further research is conducted to determine the most appropriate method of imputation for external validation in the context of survival models by comparing multiple imputation, subgroup mean imputation or sub-models using the one-step-sweep approach in a simulation study [40].

In this study, the decision was made to develop the model on the larger of the two trials causing the external validation process to be retrospective. As the two trials were conducted in the same population at different points in time, recalibration of the model coefficients and risk group boundaries was deemed unnecessary. However, as the trials’ recruiting centres largely overlapped and the test data was collected prior to the training data, this validation process was classified as retrospective rather than temporal. In addition, as myeloma outcomes have improved over time, it is likely that this decision is why, as can be seen in Additional Files 3 and 4, the calibration performance of the model is worse in the test dataset compared to the training dataset.

Before the MRP can be applied in clinic, further validation processes are required as well as an impact analysis [41]. As the MRP was trained and tested in UK clinical trial data, there is a question over its generalisability. Therefore, an external validation process will be conducted within the CoMMpass study (NCT01454297). The CoMMpass study contains real-world patients from Canada, Italy, Spain and the USA and was collected by the MMRF (Multiple Myeloma Research Foundation) independently from the UK myeloma research community. Therefore, it represents a dataset which is entirely external from the data considered here. However, the CoMMpass study represents another retrospective validation process and before the model can be used in practice, a prospective validation process should be considered. Myeloma XIV is a UK clinical trial in TNE patients with newly diagnosed multiple myeloma which opened to recruitment on the 4th August 2020 (NCT03720041). The data collected will be used in a prospective validation process, where an appropriate sample size calculation will be considered. Taiyari [42] has proposed a calculation that determines the required number of events for a prospective validation study. The calculation uses the number of events and variance of the D-statistic observed in the training dataset and calculates the required number of events in the test dataset based on the desired width and level of the confidence interval. Unsurprisingly, for the same width and level observed in the training dataset to be observed in the test dataset, the same number of events is required.

In this work, acceptable discrimination performance within both the internal and external validation processes was pre-specified. However, as it was informal and no guidance exists on how to pre-specify acceptable performance [5], it was considered problematic and removed at the request of the journal editors. A further limitation in terms of pre-specifying performance is that both discrimination and calibration must be considered. Therefore, future work is required to develop methods which can compare prognostic models in terms of both calibration and discrimination. We believe that the D-statistic is an appropriate measure for discrimination in survival models. However, calibration remains problematic. Whilst calibration slope has been investigated since this work was initially conducted, the limitations around model-misspecification and variability discussed above suggest that calibration slope may not be an appropriate measure to base calibration performance on.

Since this work was conducted two reviews of prognostic model development and validation have been published. The first considers the use of randomised controlled trial data for the development and validation of prognostic models [43]. A retrospective assessment suggests that the data was used appropriately in terms of consent, centre inclusion, eligibility and enrolment, predictor measurement, outcome and sample size. The only potential criticism was the extraneous trial effects as patients were treated, in some cases, above the standard of care. However, this will be addressed as part of the future validation processes outlined above. The second review proposes methodological standards for the application of prognostic models in clinical practice [44]. A retrospective assessment suggests that the methods applied here reflect their recommendations on the identification, development, missing data and validation of prognostic models. However, as discussed above, additional work regarding validation and impact analysis is required before the prognostic model can be applied in practice.

## Conclusion

Following guidance and examples in the literature, it was found to be possible to build a model which accounted for missing data in its development, assessment and validation processes. This has resulted in a prognostic model for overall survival obtained using a novel combination of methods that are appropriate when covariate data is missing. As discussed, this was not the only way to achieve our aim and the limitations and areas for further research have been identified.

However, this explanation of the methods provides an additional transparent example of training and testing a prognostic model for a time-to-event outcome when missing covariate data is present within the training dataset, adding to the existing literature for similar outcomes [7, 45] and a binary outcome [38].

We hope that this example motivates other researchers to have a similar open approach to reporting statistical analysis and provides additional support to those who are training and testing prognostic models in the presence of missing covariate data within the training dataset.

## Supplementary Information

**Additional file 1.** Investigations of the distributions of the potential prognostic variables LDH and CRP and implemented transformations. Histograms of the two continuous variables which were transformed as part of the preliminary investigations.

**Additional file 2.** Investigations into the correlation between each pair of potential prognostic factors. Shows the scatter plots which compared two continuous variables, the stacked bar chart which compared two categorical variables and the box plots which compared a continuous and a categorical variable from the preliminary investigations.

**Additional file 3.** Calibration assessment conducted within the training and test datasets. Shows the predicted vs observed survival probability plots calculated at 60 days and 1 year in both the training and test datasets.

**Additional file 4.** Calibration assessment conducted within the test dataset as part of the sensitivity analysis which imputed missing data in the test dataset. Shows the predicted vs observed survival probability plots calculated at 60 days and 1 year from each imputed dataset of the test data.

## Data Availability

Individual participant data (with any relevant supporting material, e.g. data dictionary, protocol, statistical analysis plan) for all trial participants (excluding any trial-specific participant opt-outs) will be made available for secondary research purposes at the end of the trial, i.e. usually when all primary and secondary endpoints have been met and all key analyses are complete. Data will be shared according to a controlled access approach, based on the following principles: ● The value of the proposal will be considered in terms of the strategic priorities of the CTRU, Chief Investigator and Sponsor, the scientific value of the proposed project, and the resources necessary and available to satisfy any data release request. ● We encourage a collaborative approach to data sharing and believe it is best practice for researchers who generated datasets to be involved in subsequent uses of those datasets. ● The timing and nature of any data release must not adversely interfere with the integrity of the trial or research project objectives, including any associated secondary and exploratory research objectives detailed in the ethically approved original research protocol. On an individual trial or research project basis, a reasonable period of exclusivity will be agreed with the trial or research project team. ● Any data release must be lawful, in line with participants’ rights and must not compromise patient confidentiality. Where the purposes of the project can be achieved by using anonymised or aggregate data, this will always be used. We will release individual patient data only in a form adjusted so that recipients of the data cannot identify individual participants by any reasonably likely means. We will also only share data when there is a binding agreement in place stating that data recipients will not attempt to re-identify any individual participants. ● Any data release must be in line with any contractual obligations to which the CTRU is subject. ● The research must be carried out by a bone fide researcher with the necessary skills and resources to conduct the research project. ● The research project must have clear objectives and use appropriate research methods. ● The research must be carried out on behalf of a reputable organisation that can demonstrate appropriate IT security standards to ensure the data is protected and to minimise the risk of unauthorised disclosure. Participants in this trial have not given explicit consent for their data to be shared for secondary research. However, they were provided with notification at trial entry of our intention to make data available for further research. In addition, data will only be made available in such a way that data recipients cannot identify individuals by any reasonably likely means, and we will only share data for projects that are clearly in the public interest and compatible with the original purpose of the data processing. Requests to access trial data should be made to CTRU-DataAccess@leeds.ac.uk in the first instance. Requests will be reviewed (based on the above principles) by relevant stakeholders. No data will be released before an appropriate agreement is in place setting out the conditions of release. The agreement will govern data retention requirements, which will usually stipulate that data recipients must delete their copy of the data at the end of the planned project. Any code mentioned in this paper can also be made available under the same process and restrictions (where applicable) as outlined above.

## Abbreviations

CI: Confidence interval
CRDa: Attenuated cyclophosphamide, lenalidomide, and dexamethasone
CRP: C-reactive protein
CTDa: Attenuated cyclophosphamide, thalidomide, and dexamethasone
CTRU: Clinical Trials Research Unit
D-statistic: Prognostic separation D-statistic
ISS: International staging system
IQR: Inter-quartile range
L:W: Ratio of lymphocytes to total white blood cells
LASSO: Least absolute shrinkage and selection operator
LDH: Lactate dehydrogenase
MAR: Missing at random
MCAR: Missing completely at random
MICE: Multiple imputation by chained equations
MMRF: Multiple Myeloma Research Foundation
MNAR: Missing not at random
MP: Melphalan and prednisolone
MRC: Medical Research Council
NCRI: National Cancer Research Institute
PS: Performance status
SD: Standard deviation
TE: Transplant eligible
TNE: Transplant ineligible
TRIPOD: Transparent reporting of a multivariable predication model for Individual Prognosis or Diagnosis
UK-MRA: United Kingdom Myeloma Research Alliance
WHO: World Health Organization
