# Supplementary material for: The development and validation of prognostic models for overall survival in the presence of missing data in the training dataset: a strategy with a detailed example
Source: Diagn Progn Res. 2021 Aug 4;5:14. doi: 10.1186/s41512-021-00103-9 (PMC8335879; doi:10.1186/s41512-021-00103-9)
Supplement: Supplementary file 1 — Additional file 1. Investigations of the distributions of the potential prognostic variables LDH and CRP and implemented transformations. Histograms of the two continuous variables which were transformed as part of the preliminary investigations. [file 41512_2021_103_MOESM1_ESM.pdf]

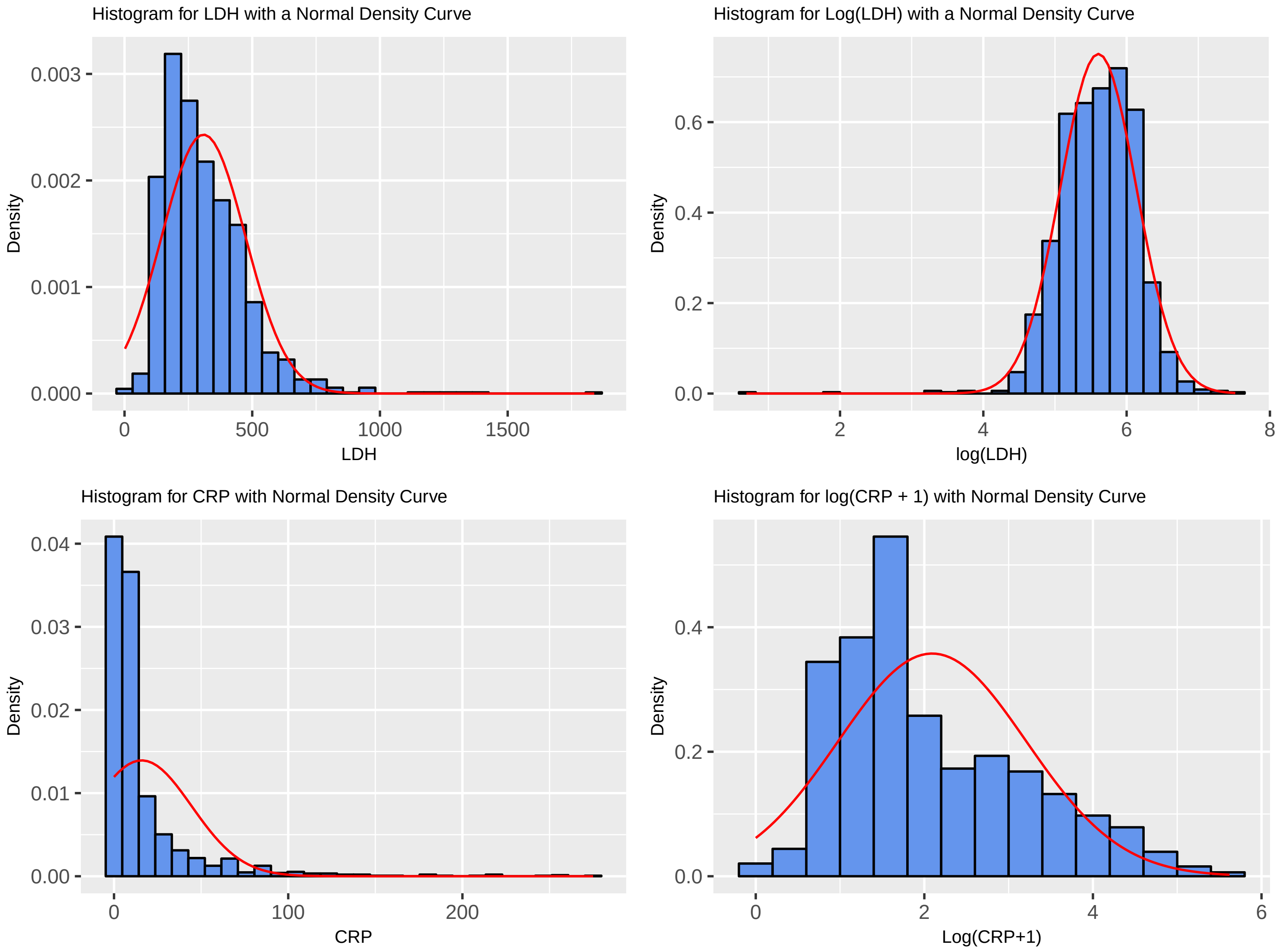

Histogram for LDH with a Normal Density Curve
Density
0.003
0.002
0.001
0.000
0
500
1000
1500
LDH
Histogram for Log(LDH) with a Normal Density Curve
Density
0.6
0.4
0.2
0.0
2
4
6
8
log(LDH)
Histogram for CRP with Normal Density Curve
Density
0.04
0.03
0.02
0.01
0.00
0
100
200
CRP
Histogram for log(CRP + 1) with Normal Density Curve
Density
0.4
0.2
0.0
0
2
4
6
Log(CRP+1)
