# Supplementary material for: The development and validation of prognostic models for overall survival in the presence of missing data in the training dataset: a strategy with a detailed example
Source: Diagn Progn Res. 2021 Aug 4;5:14. doi: 10.1186/s41512-021-00103-9 (PMC8335879; doi:10.1186/s41512-021-00103-9)
Supplement: Supplementary file 2 — Additional file 2. Investigations into the correlation between each pair of potential prognostic factors. Shows the scatter plots which compared two continuous variables, the stacked bar chart which compared two categorical variables and the box plots which compared a continuous and a categorical variable from the preliminary investigations. [file 41512_2021_103_MOESM2_ESM.pdf]

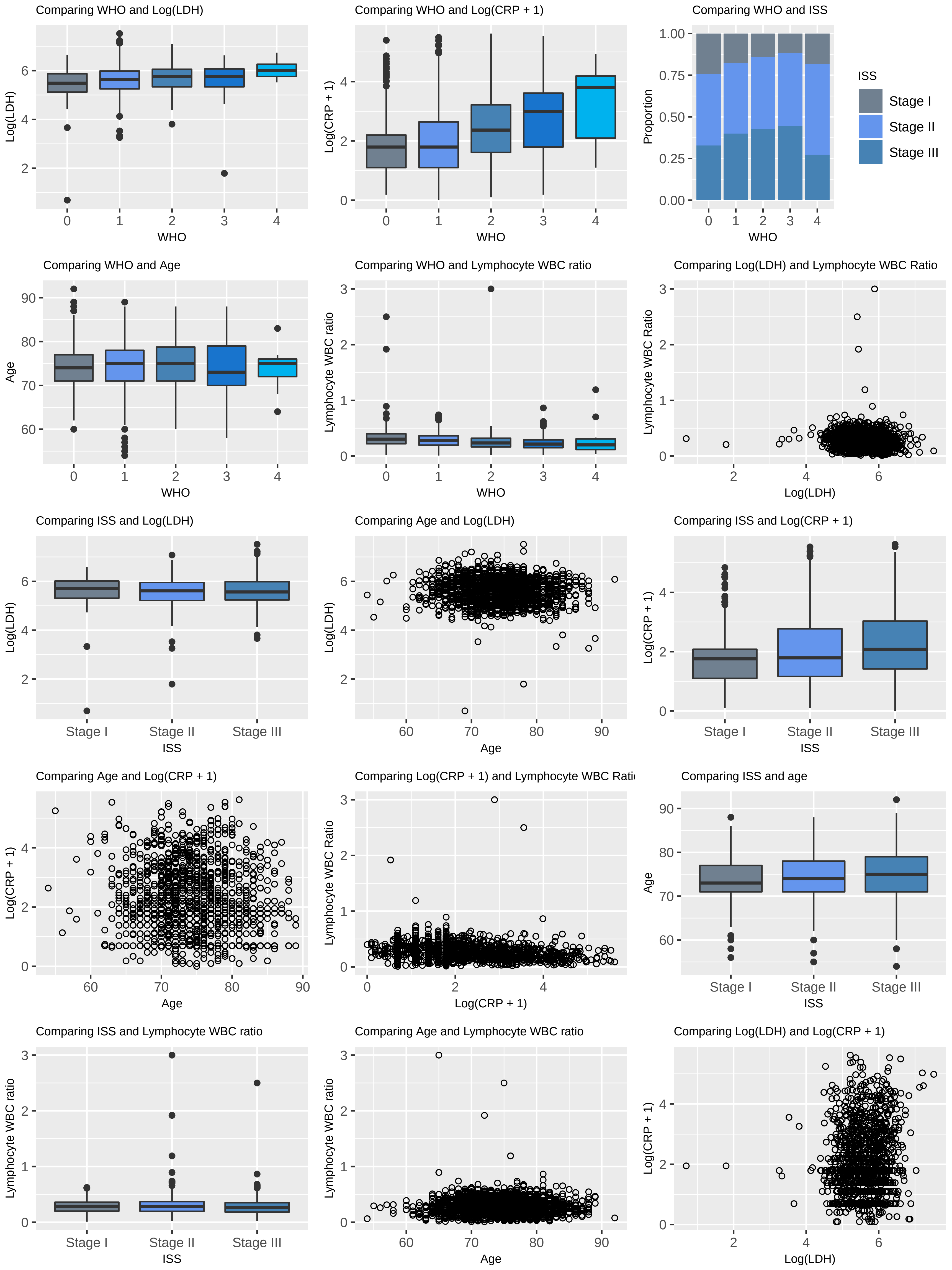

Comparing WHO and Log(LDH)
Log(LDH)
WHO
Comparing WHO and Log(CRP + 1)
Log(CRP + 1)
WHO
Comparing WHO and ISS
Proportion
WHO
ISS
Stage I
Stage II
Stage III
Comparing WHO and Age
Age
WHO
Comparing WHO and Lymphocyte WBC ratio
Lymphocyte WBC ratio
WHO
Comparing Log(LDH) and Lymphocyte WBC Ratio
Lymphocyte WBC Ratio
Log(LDH)
Comparing ISS and Log(LDH)
Log(LDH)
ISS
Comparing Age and Log(LDH)
Log(LDH)
Age
Comparing ISS and Log(CRP + 1)
Log(CRP + 1)
ISS
Comparing Age and Log(CRP + 1)
Log(CRP + 1)
Age
Comparing Log(CRP + 1) and Lymphocyte WBC Rati
Lymphocyte WBC Ratio
Log(CRP + 1)
Comparing ISS and age
Age
ISS
Comparing ISS and Lymphocyte WBC ratio
Lymphocyte WBC ratio
ISS
Comparing Age and Lymphocyte WBC ratio
Lymphocyte WBC ratio
Age
Comparing Log(LDH) and Log(CRP + 1)
Log(CRP + 1)
Log(LDH)
