# Supplementary material for: The development and validation of prognostic models for overall survival in the presence of missing data in the training dataset: a strategy with a detailed example
Source: Diagn Progn Res. 2021 Aug 4;5:14. doi: 10.1186/s41512-021-00103-9 (PMC8335879; doi:10.1186/s41512-021-00103-9)
Supplement: Supplementary file 3 — Additional file 3. Calibration assessment conducted within the training and test datasets. Shows the predicted vs observed survival probability plots calculated at 60 days and 1 year in both the training and test datasets. [file 41512_2021_103_MOESM3_ESM.pdf]

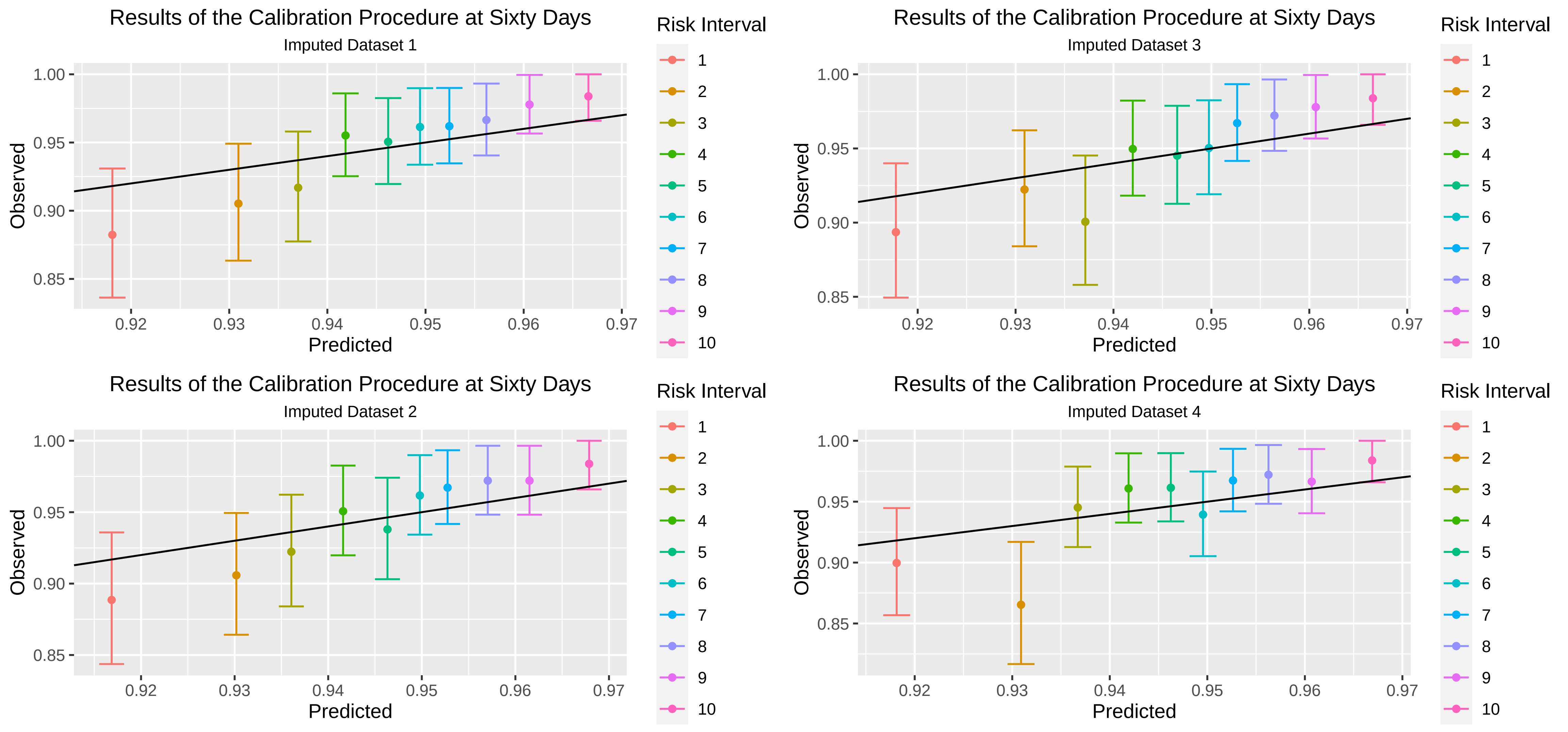
Results of the Calibration Procedure at Sixty Days
Imputed Dataset 1
Observed
Predicted
1.00
0.95
0.90
0.85
0.92
0.93
0.94
0.95
0.96
0.97
Risk Interval
1
2
3
4
5
6
7
8
9
10
Results of the Calibration Procedure at Sixty Days
Imputed Dataset 3
Observed
Predicted
1.00
0.95
0.90
0.85
0.92
0.93
0.94
0.95
0.96
0.97
Risk Interval
1
2
3
4
5
6
7
8
9
10
Results of the Calibration Procedure at Sixty Days
Imputed Dataset 2
Observed
Predicted
1.00
0.95
0.90
0.85
0.92
0.93
0.94
0.95
0.96
0.97
Risk Interval
1
2
3
4
5
6
7
8
9
10
Results of the Calibration Procedure at Sixty Days
Imputed Dataset 4
Observed
Predicted
1.00
0.95
0.90
0.85
0.92
0.93
0.94
0.95
0.96
0.97
Risk Interval
1
2
3
4
5
6
7
8
9
10

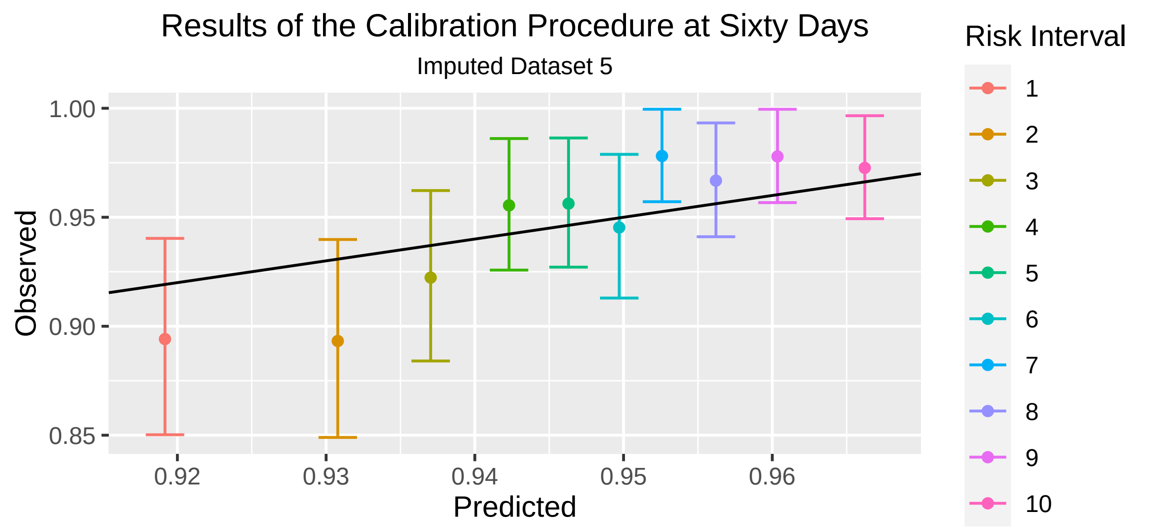
Results of the Calibration Procedure at Sixty Days
Imputed Dataset 5
Observed
1.00
0.95
0.90
0.85
0.92
0.93
0.94
0.95
0.96
Predicted
Risk Interval
1
2
3
4
5
6
7
8
9
10

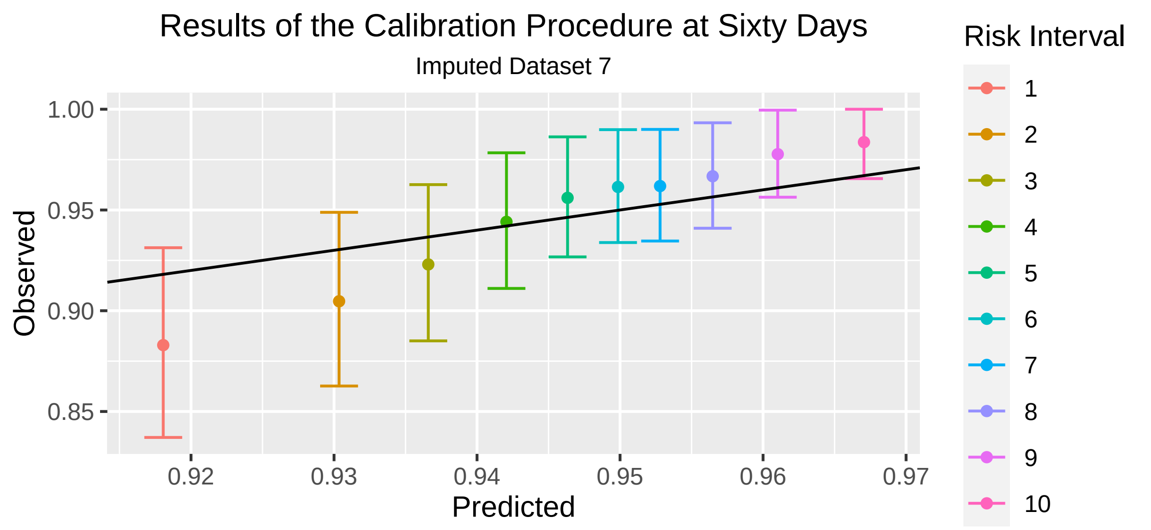
Results of the Calibration Procedure at Sixty Days
Imputed Dataset 7
Observed
1.00
0.95
0.90
0.85
0.92
0.93
0.94
0.95
0.96
0.97
Predicted
Risk Interval
1
2
3
4
5
6
7
8
9
10

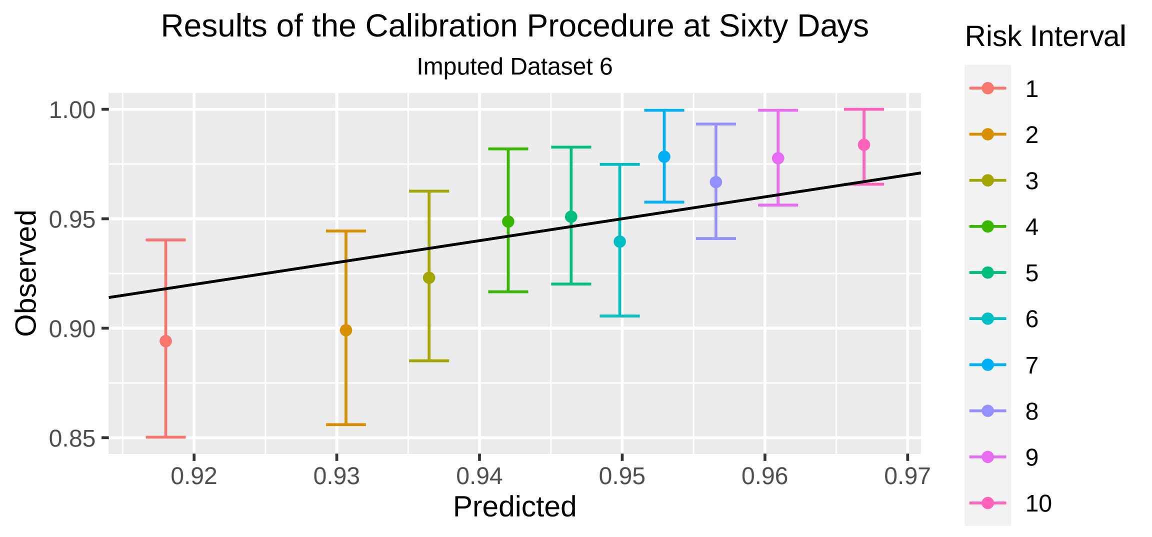
Results of the Calibration Procedure at Sixty Days
Imputed Dataset 6
Observed
1.00
0.95
0.90
0.85
0.92
0.93
0.94
0.95
0.96
0.97
Predicted
Risk Interval
1
2
3
4
5
6
7
8
9
10

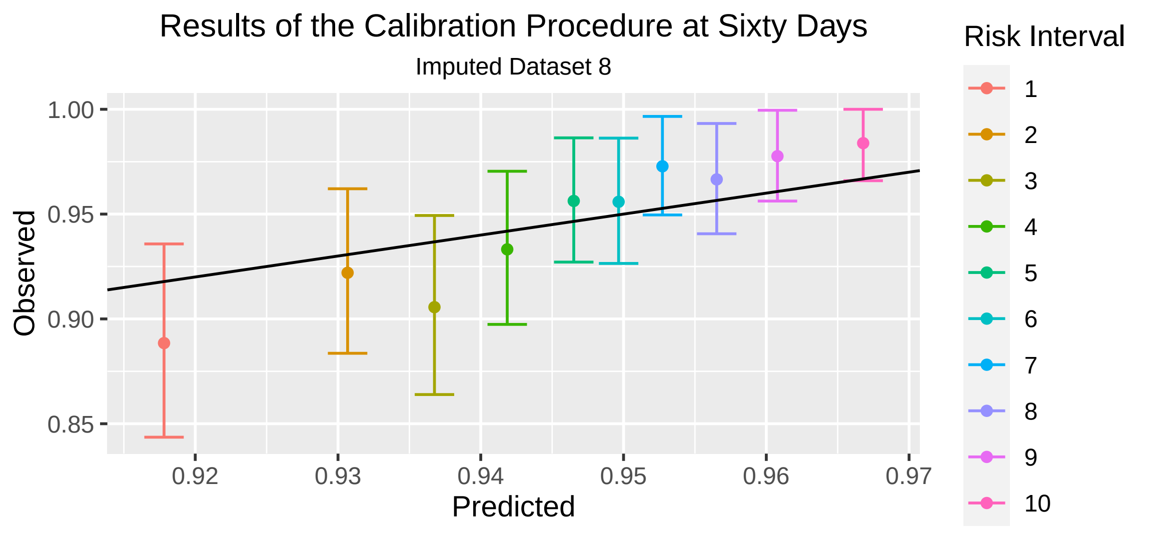
Results of the Calibration Procedure at Sixty Days
Imputed Dataset 8
Observed
1.00
0.95
0.90
0.85
0.92
0.93
0.94
0.95
0.96
0.97
Predicted
Risk Interval
1
2
3
4
5
6
7
8
9
10

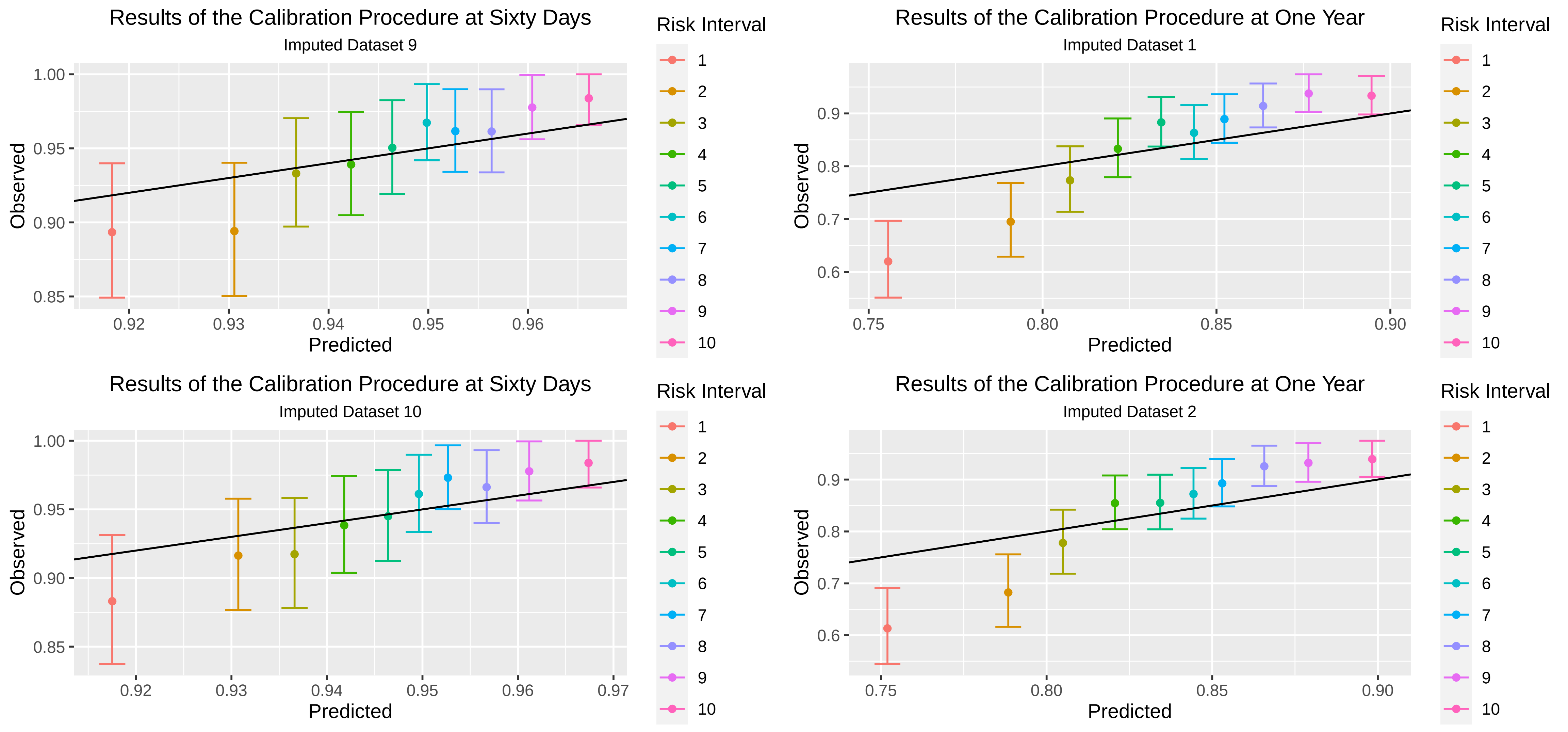
Results of the Calibration Procedure at Sixty Days
Imputed Dataset 9
Observed
Predicted
1.00
0.95
0.90
0.85
0.92
0.93
0.94
0.95
0.96
Risk Interval
1
2
3
4
5
6
7
8
9
10
Results of the Calibration Procedure at One Year
Imputed Dataset 1
Observed
Predicted
0.9
0.8
0.7
0.6
0.75
0.80
0.85
0.90
Risk Interval
1
2
3
4
5
6
7
8
9
10
Results of the Calibration Procedure at Sixty Days
Imputed Dataset 10
Observed
Predicted
1.00
0.95
0.90
0.85
0.92
0.93
0.94
0.95
0.96
0.97
Risk Interval
1
2
3
4
5
6
7
8
9
10
Results of the Calibration Procedure at One Year
Imputed Dataset 2
Observed
Predicted
0.9
0.8
0.7
0.6
0.75
0.80
0.85
0.90
Risk Interval
1
2
3
4
5
6
7
8
9
10

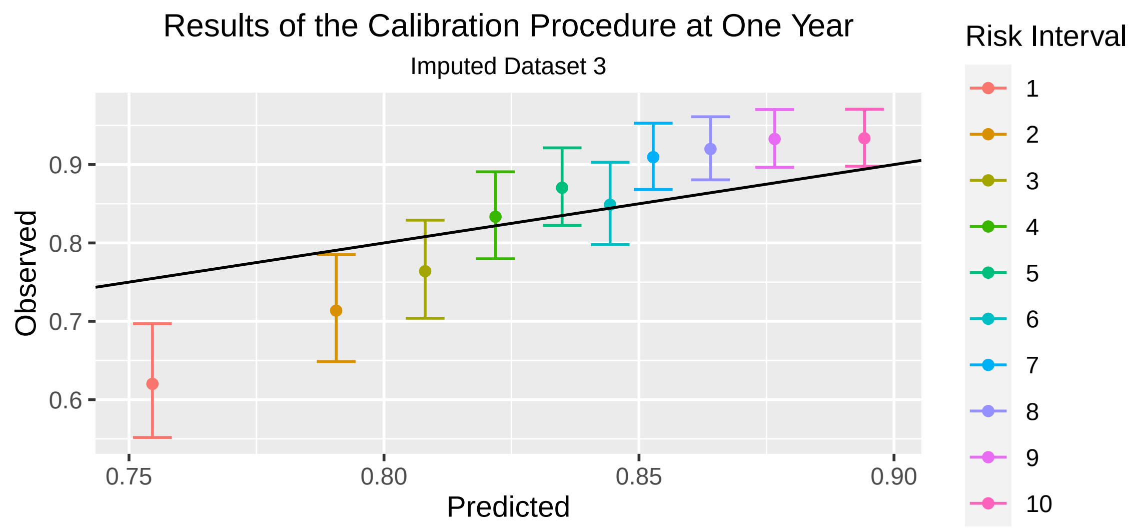
Results of the Calibration Procedure at One Year
Imputed Dataset 3
Observed
Predicted
0.6
0.7
0.8
0.9
0.75
0.80
0.85
0.90
Risk Interval
1
2
3
4
5
6
7
8
9
10

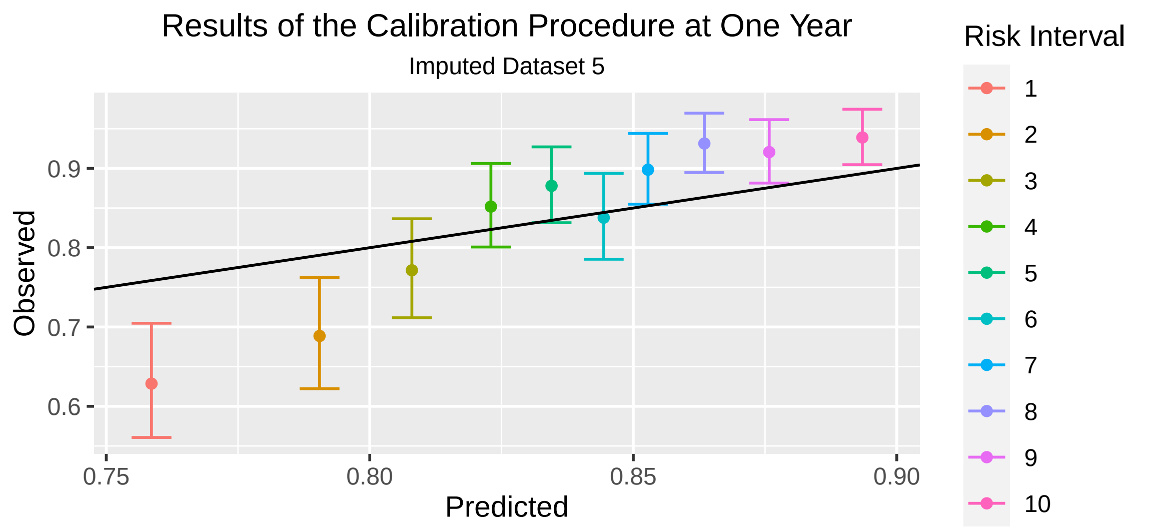
Results of the Calibration Procedure at One Year
Imputed Dataset 5
Observed
Predicted
0.6
0.7
0.8
0.9
0.75
0.80
0.85
0.90
Risk Interval
1
2
3
4
5
6
7
8
9
10

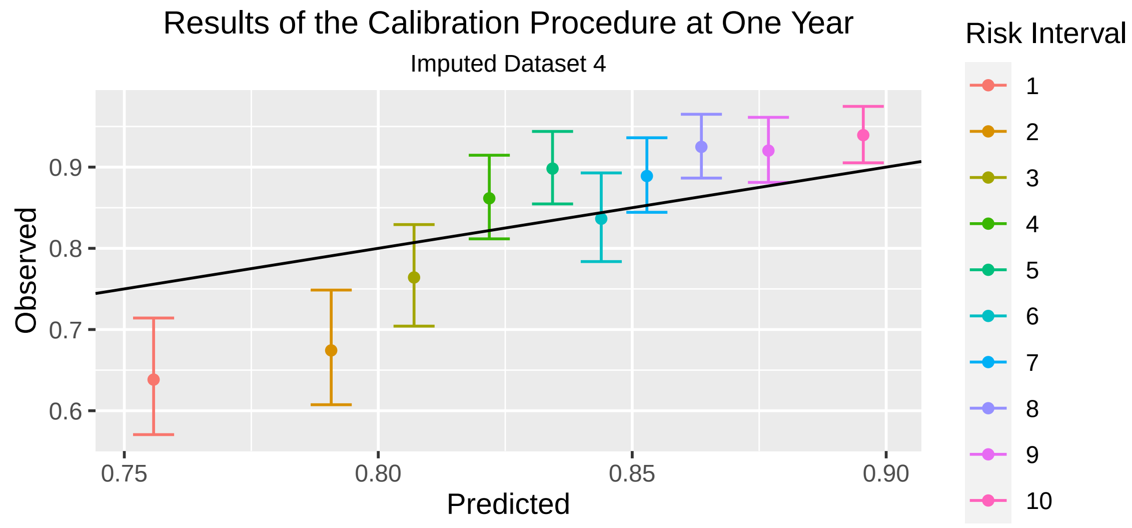
Results of the Calibration Procedure at One Year
Imputed Dataset 4
Observed
Predicted
0.6
0.7
0.8
0.9
0.75
0.80
0.85
0.90
Risk Interval
1
2
3
4
5
6
7
8
9
10

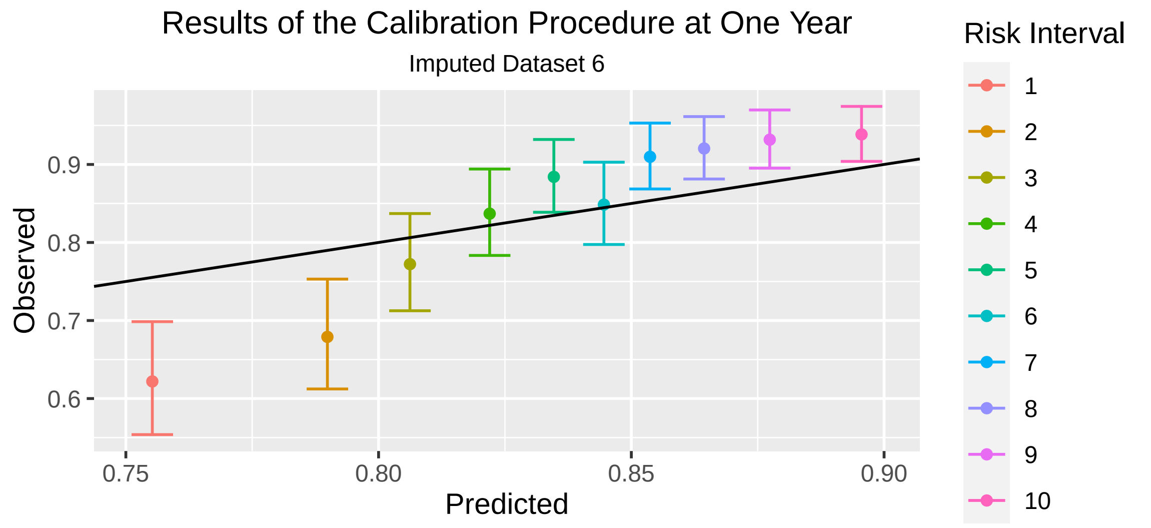
Results of the Calibration Procedure at One Year
Imputed Dataset 6
Observed
Predicted
0.6
0.7
0.8
0.9
0.75
0.80
0.85
0.90
Risk Interval
1
2
3
4
5
6
7
8
9
10

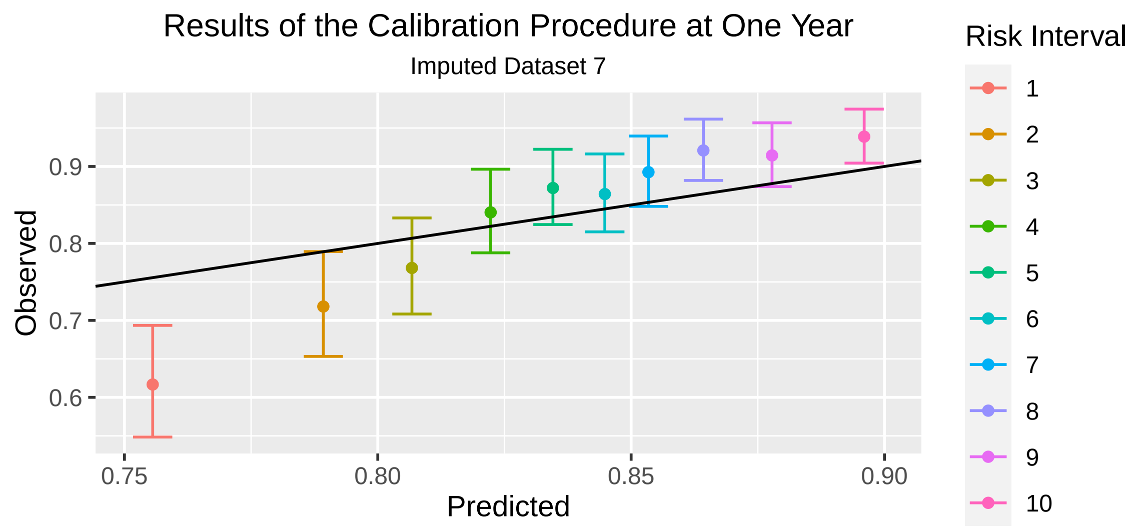
Results of the Calibration Procedure at One Year
Imputed Dataset 7
Observed
Predicted
0.6
0.7
0.8
0.9
0.75
0.80
0.85
0.90
Risk Interval
1
2
3
4
5
6
7
8
9
10

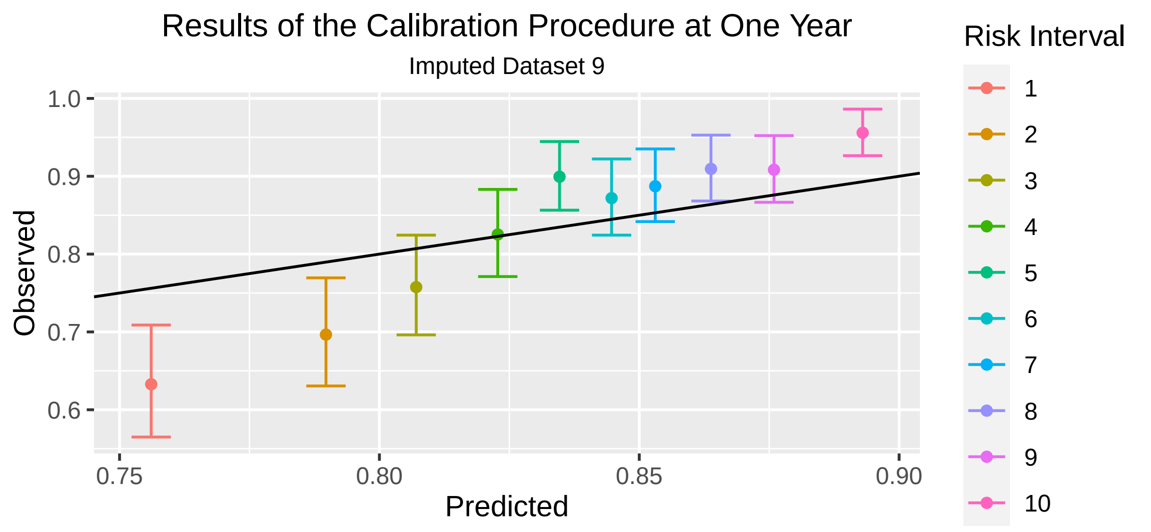
Results of the Calibration Procedure at One Year
Imputed Dataset 9
Observed
Predicted
0.6
0.7
0.8
0.9
1.0
0.75
0.80
0.85
0.90
Risk Interval
1
2
3
4
5
6
7
8
9
10

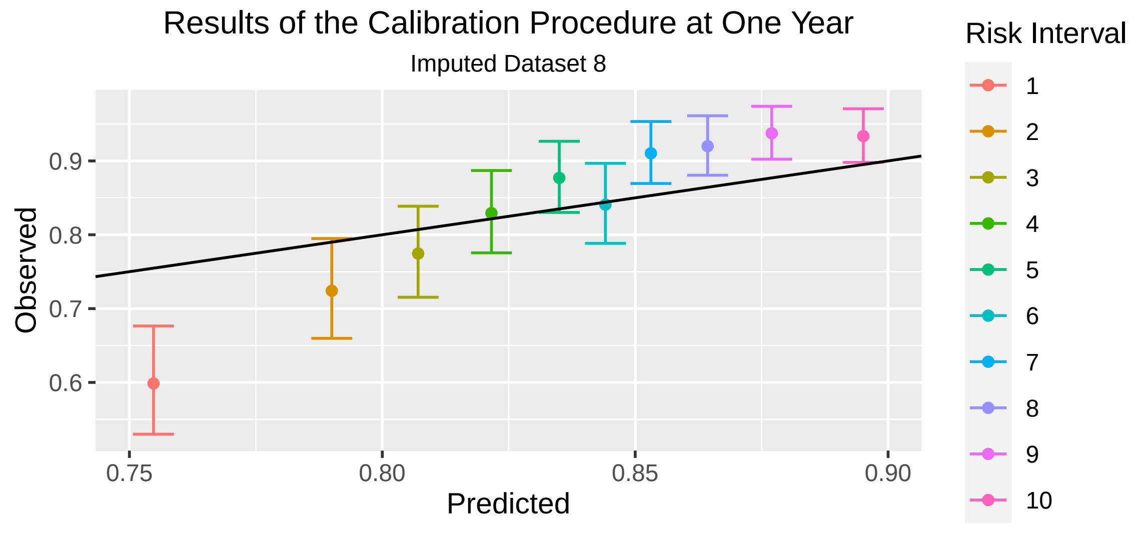
Results of the Calibration Procedure at One Year
Imputed Dataset 8
Observed
Predicted
0.6
0.7
0.8
0.9
0.75
0.80
0.85
0.90
Risk Interval
1
2
3
4
5
6
7
8
9
10

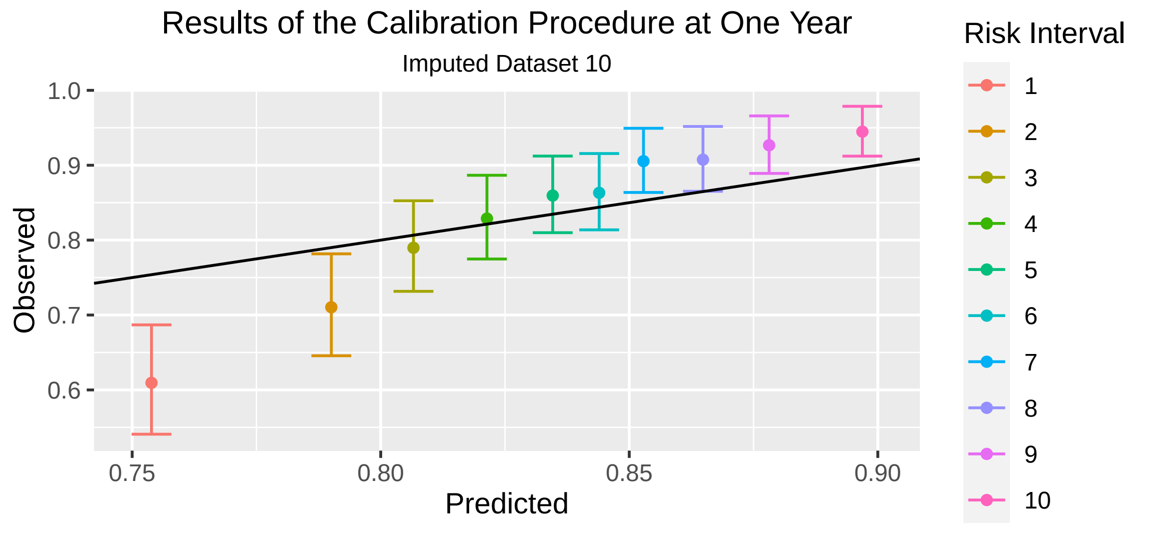
Results of the Calibration Procedure at One Year
Imputed Dataset 10
Observed
Predicted
0.6
0.7
0.8
0.9
1.0
0.75
0.80
0.85
0.90
Risk Interval
1
2
3
4
5
6
7
8
9
10

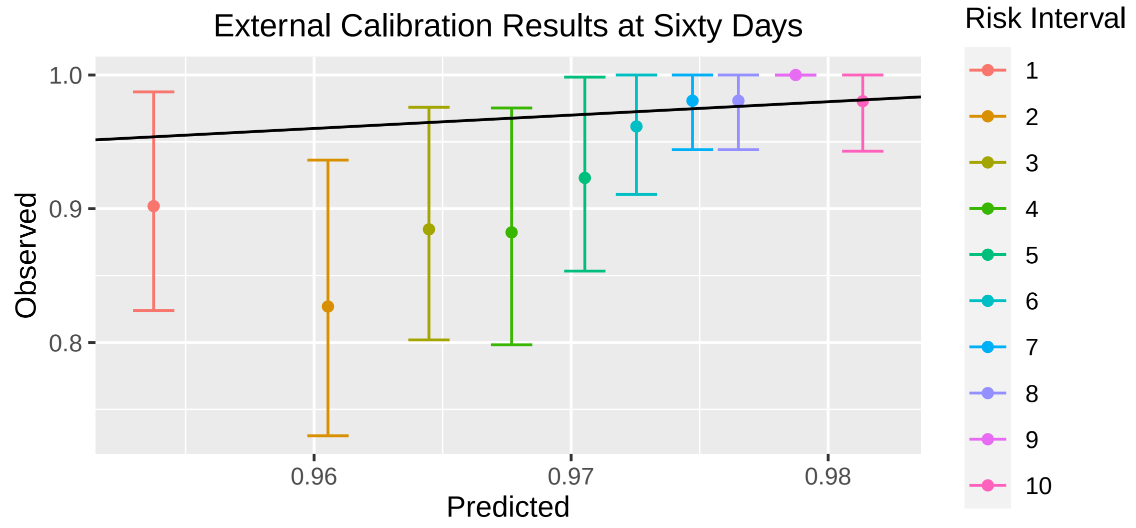
External Calibration Results at Sixty Days
Observed
Predicted
1.0
0.9
0.8
0.96
0.97
0.98
Risk Interval
1
2
3
4
5
6
7
8
9
10

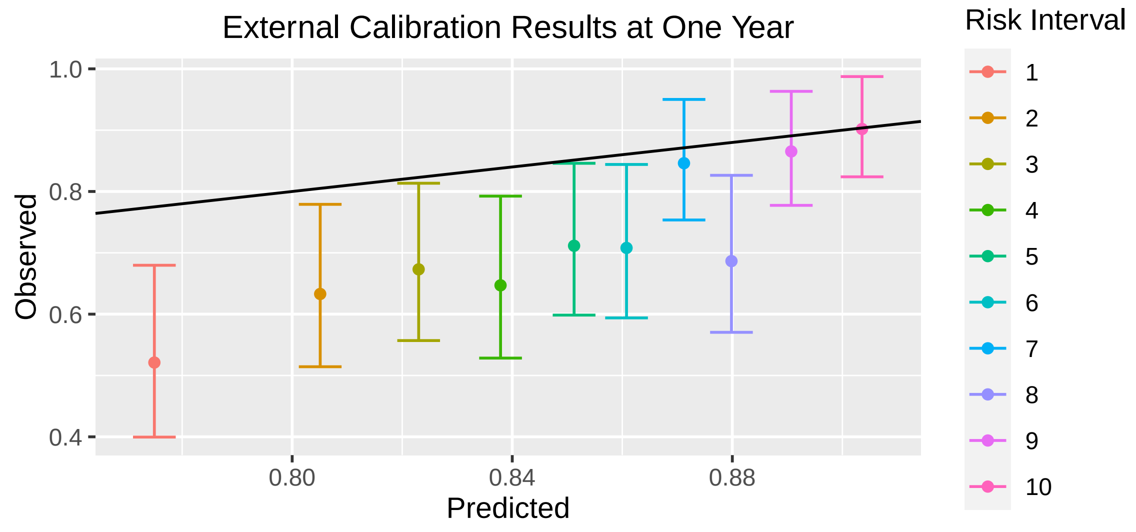
External Calibration Results at One Year
Observed
Predicted
1.0
0.8
0.6
0.4
0.80
0.84
0.88
Risk Interval
1
2
3
4
5
6
7
8
9
10
