# Supplementary material for: The development and validation of prognostic models for overall survival in the presence of missing data in the training dataset: a strategy with a detailed example
Source: Diagn Progn Res. 2021 Aug 4;5:14. doi: 10.1186/s41512-021-00103-9 (PMC8335879; doi:10.1186/s41512-021-00103-9)
Supplement: Supplementary file 4 — Additional file 4. Calibration assessment conducted within the test dataset as part of the sensitivity analysis which imputed missing data in the test dataset. Shows the predicted vs observed survival probability plots calculated at 60 days and 1 year from each imputed dataset of the test data. [file 41512_2021_103_MOESM4_ESM.pdf]

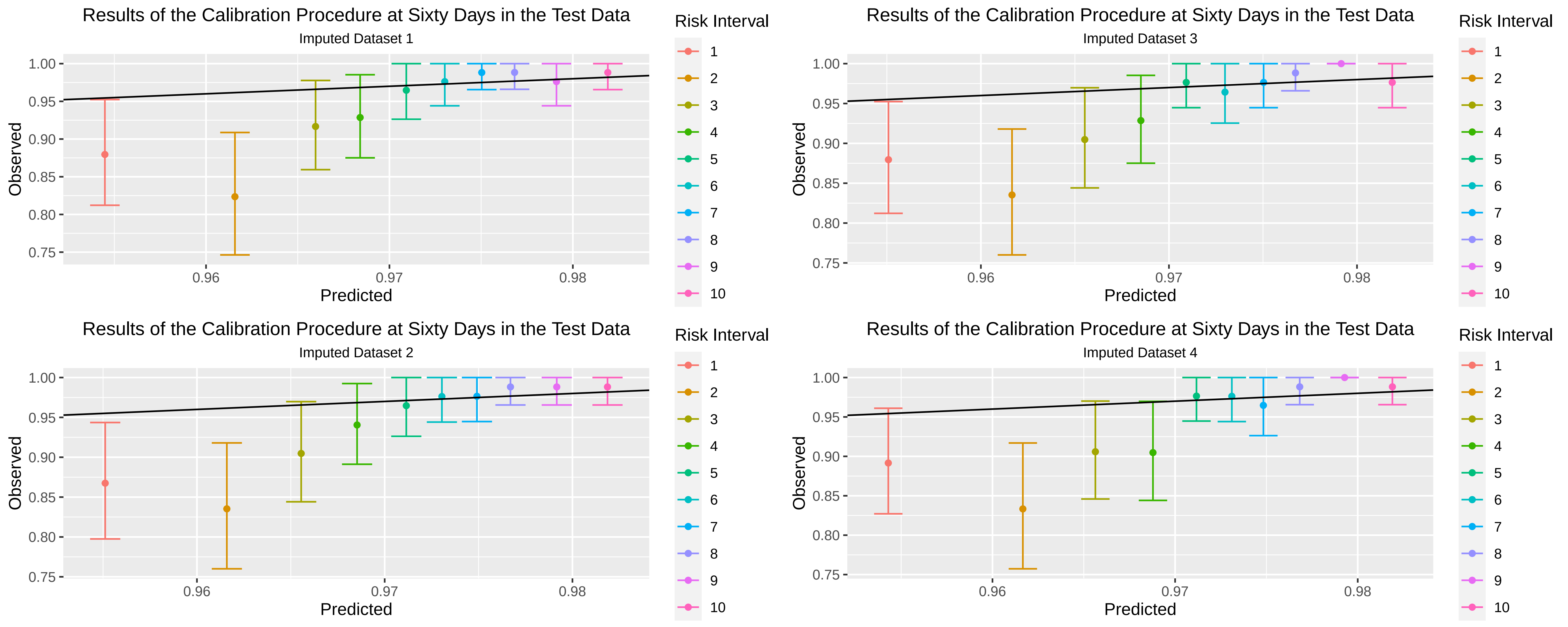
Results of the Calibration Procedure at Sixty Days in the Test Data
Imputed Dataset 1
Imputed Dataset 2
Imputed Dataset 3
Imputed Dataset 4
Observed
Predicted
1.00
0.95
0.90
0.85
0.80
0.75
0.96
0.97
0.98
Risk Interval
1
2
3
4
5
6
7
8
9
10

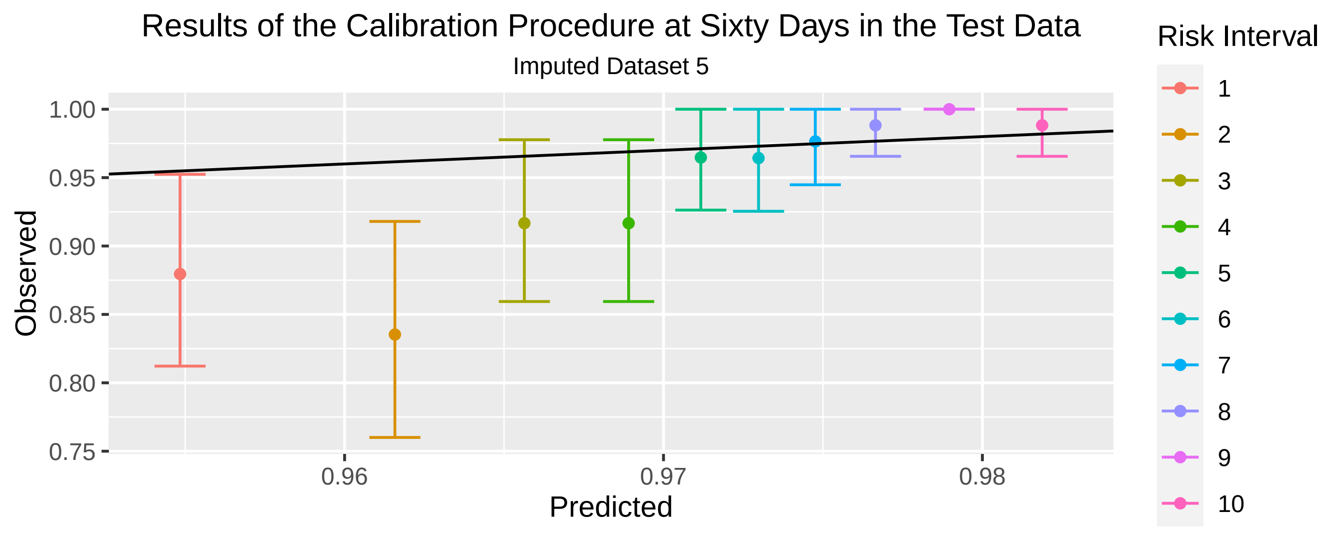
Results of the Calibration Procedure at Sixty Days in the Test Data
Imputed Dataset 5
Observed
Predicted
1.00
0.95
0.90
0.85
0.80
0.75
0.96
0.97
0.98
Risk Interval
1
2
3
4
5
6
7
8
9
10

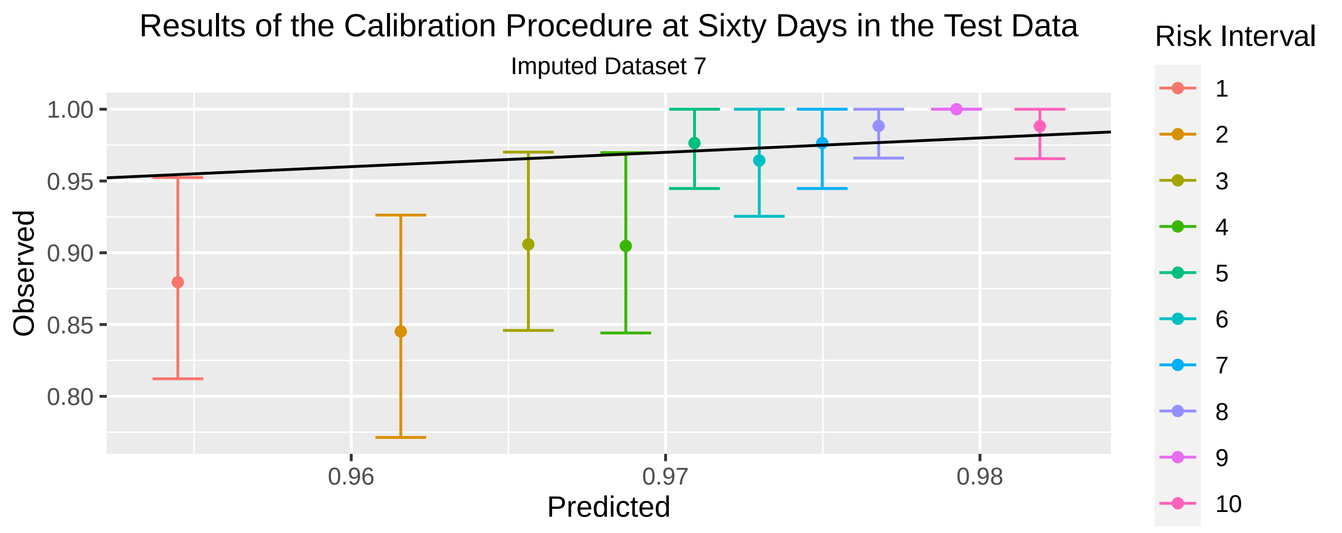
Results of the Calibration Procedure at Sixty Days in the Test Data
Imputed Dataset 7
Observed
Predicted
1.00
0.95
0.90
0.85
0.80
0.96
0.97
0.98
Risk Interval
1
2
3
4
5
6
7
8
9
10

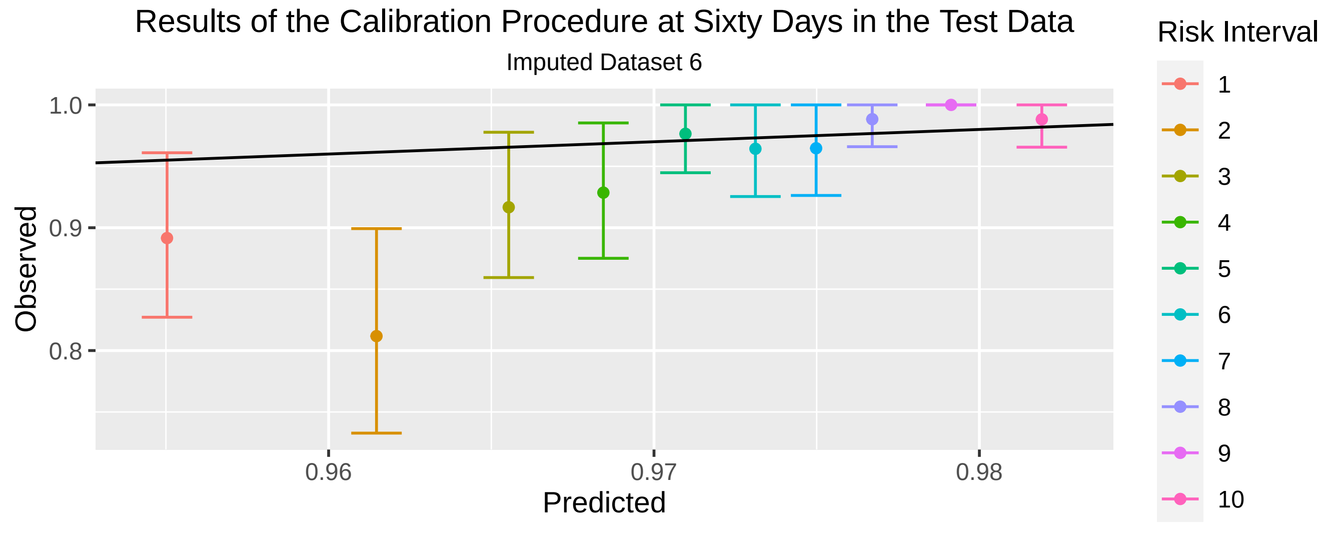
Results of the Calibration Procedure at Sixty Days in the Test Data
Imputed Dataset 6
Observed
Predicted
1.0
0.9
0.8
0.96
0.97
0.98
Risk Interval
1
2
3
4
5
6
7
8
9
10

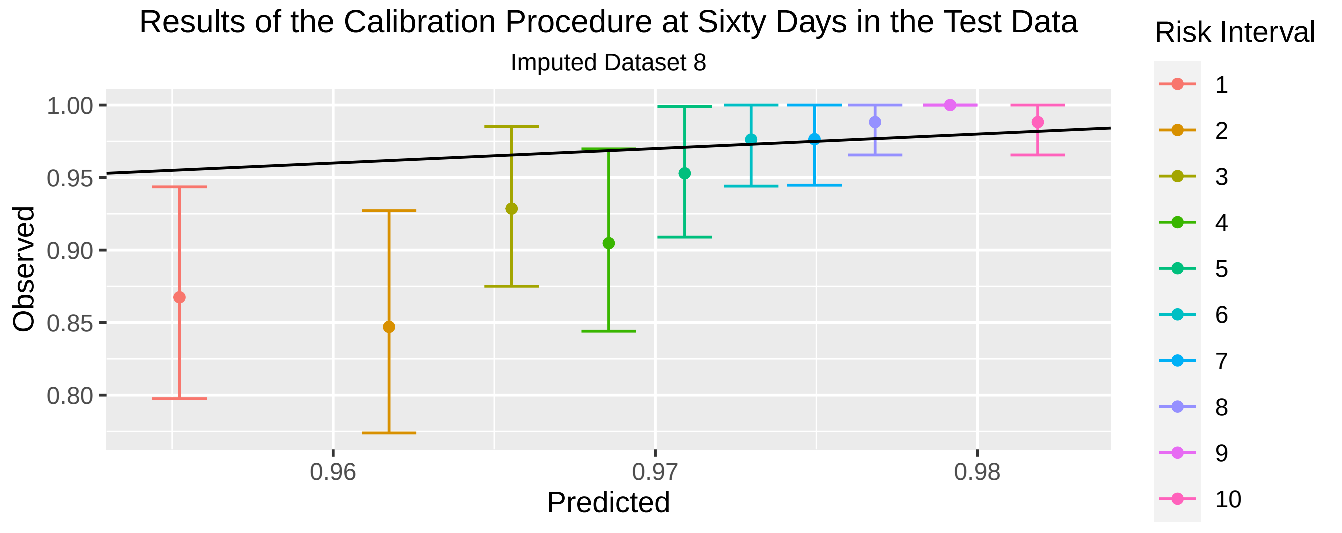
Results of the Calibration Procedure at Sixty Days in the Test Data
Imputed Dataset 8
Observed
Predicted
1.00
0.95
0.90
0.85
0.80
0.96
0.97
0.98
Risk Interval
1
2
3
4
5
6
7
8
9
10

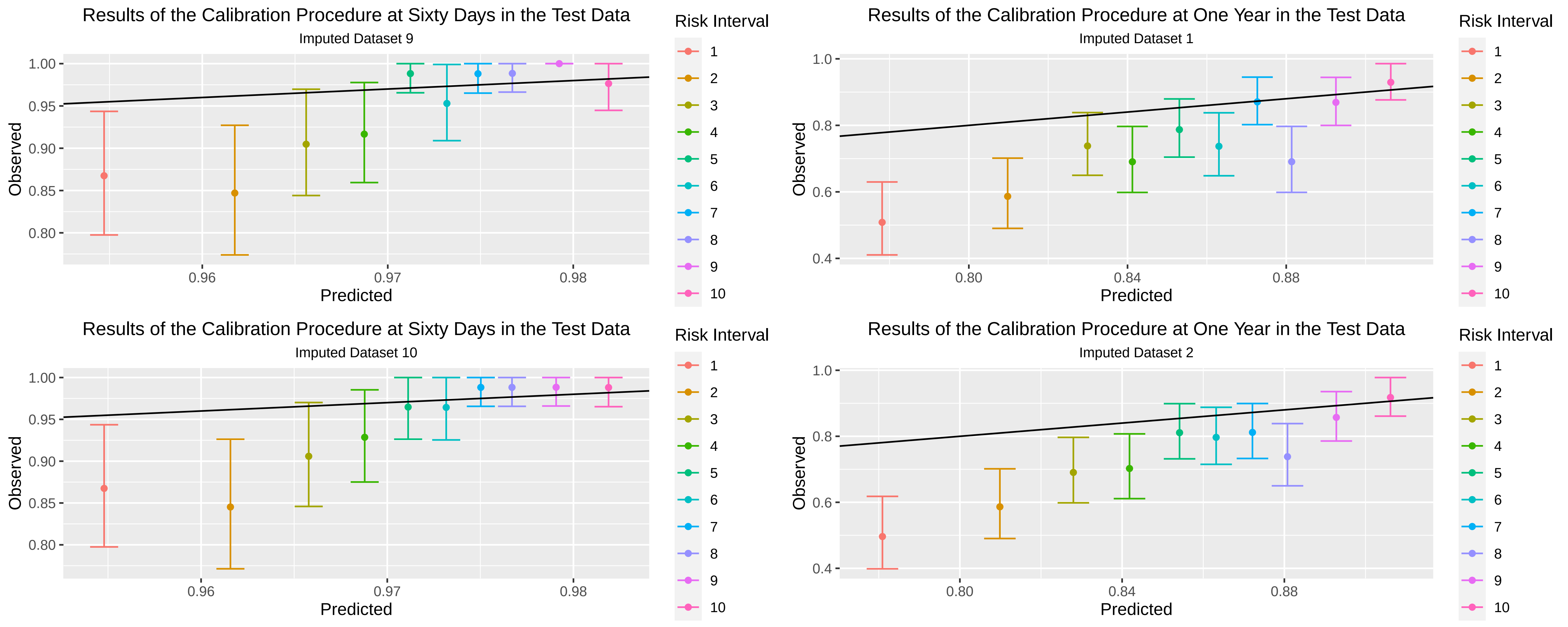
Results of the Calibration Procedure at Sixty Days in the Test Data
Imputed Dataset 9
Observed
Predicted
1.00
0.95
0.90
0.85
0.80
0.96
0.97
0.98
Risk Interval
1
2
3
4
5
6
7
8
9
10
Results of the Calibration Procedure at One Year in the Test Data
Imputed Dataset 1
Observed
Predicted
1.0
0.8
0.6
0.4
0.80
0.84
0.88
Risk Interval
1
2
3
4
5
6
7
8
9
10
Results of the Calibration Procedure at Sixty Days in the Test Data
Imputed Dataset 10
Observed
Predicted
1.00
0.95
0.90
0.85
0.80
0.96
0.97
0.98
Risk Interval
1
2
3
4
5
6
7
8
9
10
Results of the Calibration Procedure at One Year in the Test Data
Imputed Dataset 2
Observed
Predicted
1.0
0.8
0.6
0.4
0.80
0.84
0.88
Risk Interval
1
2
3
4
5
6
7
8
9
10

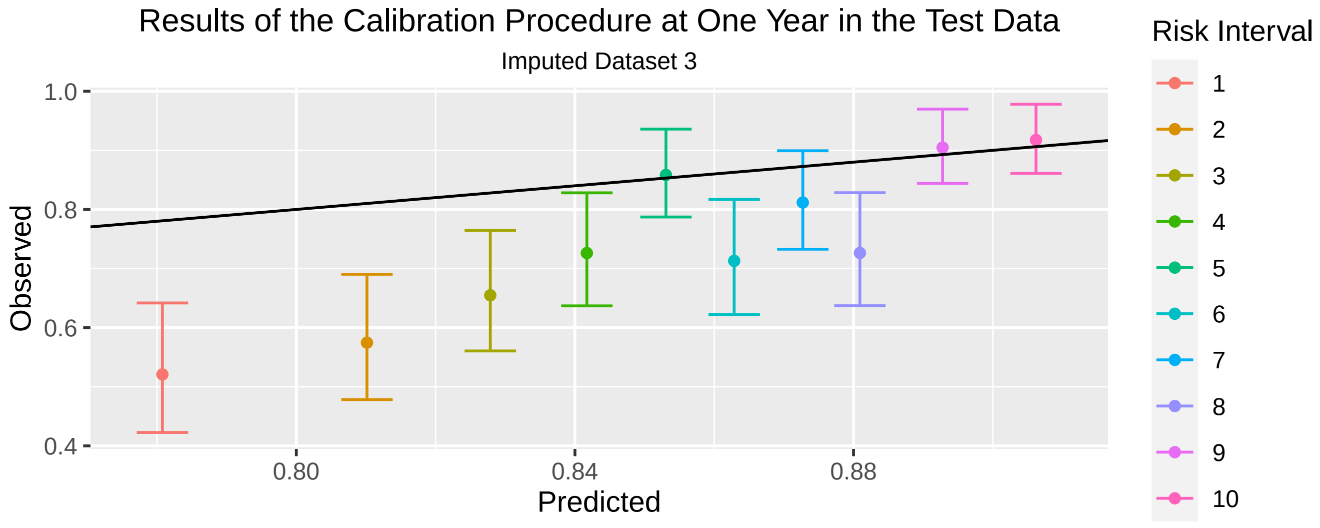
Results of the Calibration Procedure at One Year in the Test Data
Imputed Dataset 3
Observed
Predicted
Risk Interval
1
2
3
4
5
6
7
8
9
10

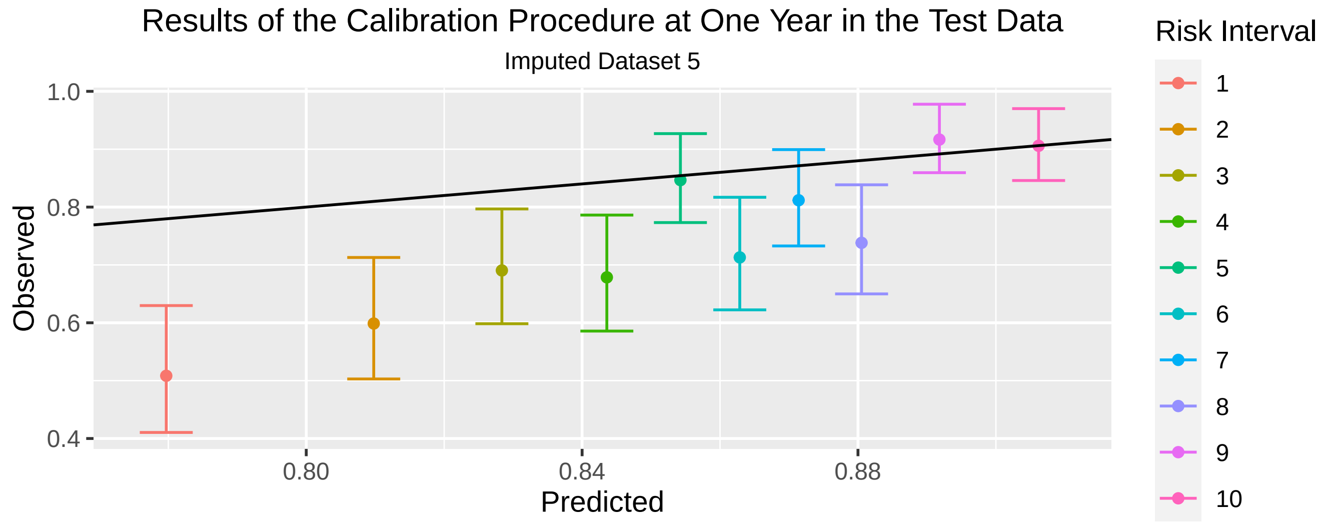
Results of the Calibration Procedure at One Year in the Test Data
Imputed Dataset 5
Observed
Predicted
Risk Interval
1
2
3
4
5
6
7
8
9
10

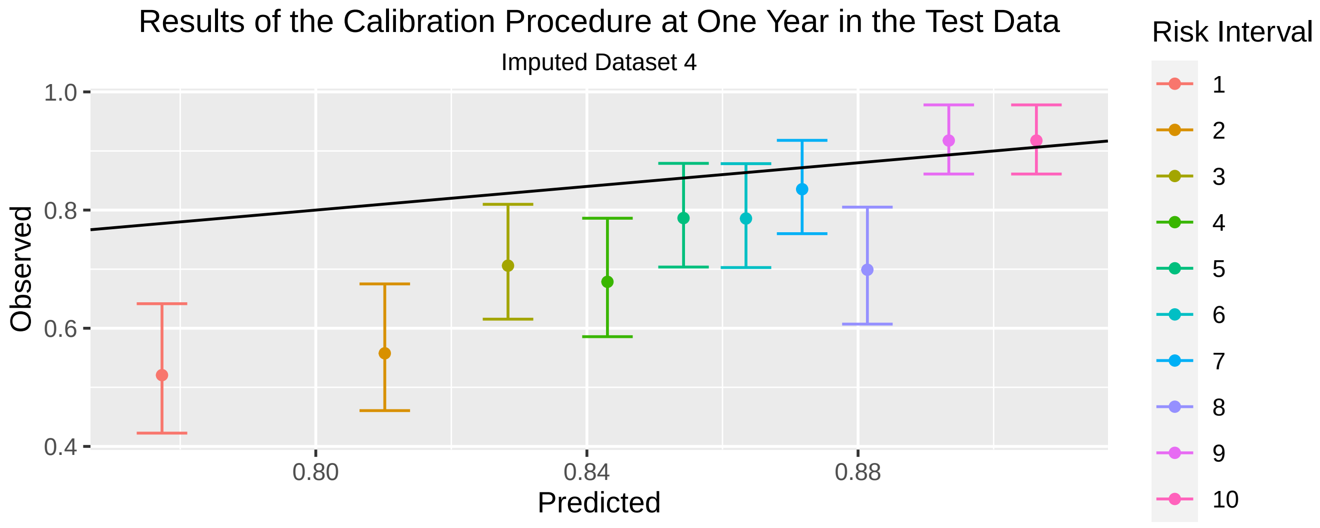
Results of the Calibration Procedure at One Year in the Test Data
Imputed Dataset 4
Observed
Predicted
Risk Interval
1
2
3
4
5
6
7
8
9
10

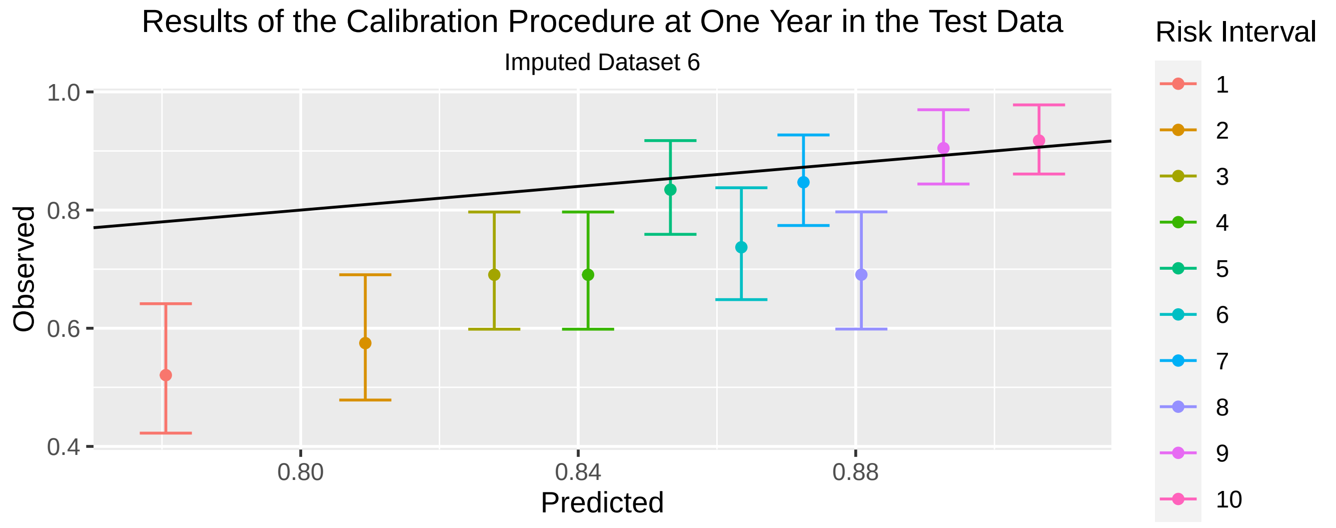
Results of the Calibration Procedure at One Year in the Test Data
Imputed Dataset 6
Observed
Predicted
Risk Interval
1
2
3
4
5
6
7
8
9
10

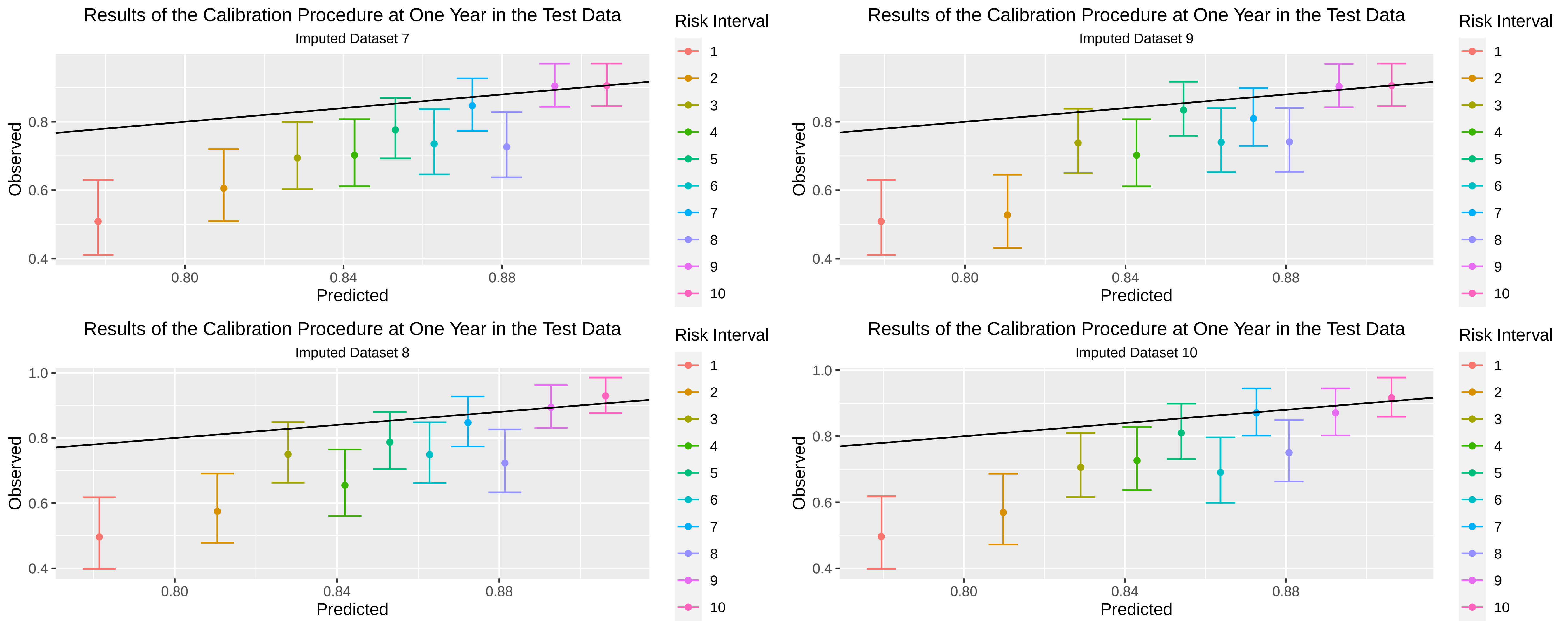
Results of the Calibration Procedure at One Year in the Test Data
Imputed Dataset 7
Imputed Dataset 9
Imputed Dataset 8
Imputed Dataset 10
Risk Interval
1
2
3
4
5
6
7
8
9
10
Observed
Predicted
0.4
0.6
0.8
1.0
0.80
0.84
0.88
